# Real world clinical feasibility of direct-from-specimen antimicrobial susceptibility testing of clinical specimens with unknown microbial load or susceptibility

**DOI:** 10.1038/s41598-022-21970-2

**Published:** 2022-11-02

**Authors:** Jade Chen, Eduardo Navarro, Brian Mesich, Derek Gerstbrein, Amorina Cruz, Matthew L. Faron, Vincent Gau

**Affiliations:** 1 GeneFluidics, Los Angeles, CA USA; 2 The Medical College of Wisconsin, Milwaukee, WI USA

**Keywords:** Clinical microbiology, Antimicrobial resistance

## Abstract

Within healthcare settings, physicians use antibiograms, which offer information on local susceptibility rates, as an aid in selecting empirical antibiotic therapy and avoiding the prescription of potentially ineffective drugs. While antibiograms display susceptibility and resistance data at hospital, city, or region-specific levels and ultimately enable the initiation of antibiogram-based empirical antibiotic treatment, AST reports at the individual patient level and guides treatments away from broad-spectrum antibiotics towards narrower-spectrum antibiotics or the removal of antibiotics entirely. Despite these advantages, AST traditionally requires a 48- to 72-h turn-around; this window of time can be critical for some antimicrobial therapeutic interventions. Herein, we present a direct-from-specimen AST to reduce the time between patient sampling and receipt of lab AST results. The biggest challenge of performing AST directly from unprocessed clinical specimens with an unknown microbial load is aligning the categorical susceptibility report with CLSI reference methods, which start from a fixed inoculum of 0.5 McFarland units prepared using colonies from a sub-culture. In this pilot clinical feasibility study using de-identified remnant specimens collected from MCW, we observed the high and low ends of microbial loads, demonstrating a final categorical agreement of 87.5% for ampicillin, 100% for ciprofloxacin, and 100% for sulfamethoxazole-trimethoprim.

## Introduction

Conventional clinical microbiology is typically based on either single parameter reporting, such as the diameter of the zone of inhibition reported in disk diffusion antimicrobial susceptibility testing (AST), or complex manual interpretation, such as the morphological analysis of microscopic plate images of colonies for pathogen identification. While both methods are considered gold standards, there is a clear preference for rapid testing. However, a major constraint of rapid diagnostics is the analysis required from a skilled specialist to gain meaningful information from microbiological assays. Herein, we present the development of a microbiological assay intended to establish a microbial growth response curve over a spectrum of microbial-to-antimicrobial ratios covering potential specimen conditions, after which the quantifiable characteristics of the curve can be digitized for fully automated analysis with a reporting algorithm. Artificial intelligence (AI) has increasingly been used to develop standard of care by identifying high-impact diseases with established medical practice patterns; antibiogram-based personalized, or targeted, antimicrobial therapy is no exception^1,2^. Big data methods from electronic health record systems are being broadly adopted into healthcare systems for population and evidence-based clinical decision-making. Alongside these methods, AI techniques and guidelines established by infectious disease specialists may help overcome the challenges limiting individualized patient care by ruling out antibiotics deemed potentially ineffective due to hospital-specific emerging antimicrobial resistance. Patients vary widely with respect to infectious disease causative pathogens and treatment responsiveness; therefore, appropriate intervention targets and strategies for personalizing medicine with AST have been adopted as one of the earliest forms of personalized medicine, providing predictive information on specific antibiotics for treatment of a patient's infection^3^. However, the ability of AI to advance personalized medicine is severely limited by the availability of timely assays and methods of accessing and ultimately integrating the personalized responses into the population-based big data. As stated by Dr. She from USC Keck School of Medicine, although innovations in direct-from-specimen pathogen identification and AST have been reported for decades, these methods have not been widely adopted into clinical practice, primarily due to workflow and practicality considerations, as well as concerns regarding the analytical performance characteristics of these methods with respect to the current reference methods^4^. The paradigm shift in phenotypic AST from starting with isolates of an overnight sub-culture to starting directly from unprocessed clinical specimens is inevitable in the effort to provide timely precision treatment with high levels of accuracy and interpretability.

In following institutional antimicrobial stewardship, a clinician may wait for diagnoses before prescribing treatment, ultimately risking a patient’s chances of recovery. To avoid doing so, the clinician is often required to make a guided decision to initiate empiric antibiotic therapy without knowing the lab results. Ideally, rapid diagnoses would be available within 8 h, the length of a hospital shift. As published previously, the challenges of developing a direct-from-specimen AST to be performed in 8 h or less are (1) unknown microbial load, (2) polymicrobial versus monomicrobial populations, and (3) equivalence in reporting to CLSI reference methods^5^. The goal of the patient-specific direct-from-specimen AST is to rule out ineffective antibiotics similarly to the population-based antibiogram. Here, we present a real-world clinical feasibility study to demonstrate the implementation of a direct-from-specimen AST into clinical routines and to exhibit agreement with CLSI reference methods. The specimen transportation time, as previously published, may range from less than an hour for an in-hospital clinical microbiological laboratory to approximately 20 h for an overnight shipment to a centralized laboratory^6^. In the presented study, we focus only on overnight shipments and do not consider this shipment time as part of the direct-from-specimen AST assay time.

## Materials and methods

### Optimization studies with remnant specimens from MCW

The signal resolution of each end of the clinically relevant microbial load range for inpatient and outpatient settings is easily affected by different collection and testing parameters. The presented direct-from-specimen AST was optimized by assessing the impact of skewed response curves from specimens with high microbial loads, the throughput and cost of goods sold, and the suppressed change in inhibited growth. Testing conditions were evaluated with de-identified remnant urine specimens shipped from the Medical College of Wisconsin (MCW); categorical agreement was calculated by comparing assay and disk diffusion results.

### Direct-from-specimen AST clinical feasibility study

After completing the optimization studies, the presented clinical feasibility study was performed using de-identified remnant clinical urine specimens shipped from MCW. Specimen dilution and starting volume were studied to address the limited dynamic range of the response curves observed during optimization. Specimen dilution concentration may be the most important variable in AST, as using a much higher or lower concentration may skew the resulting minimum inhibitory concentration (MIC): higher MIC for high specimen concentration, lower MIC for low concentration^7^. For this reason, when determining susceptibility using the CLSI susceptibility breakpoints, it is recommended to use an inoculum of 5 × 10^5^ CFU/mL for the broth microdilution method. Given the challenge of an unknown microbial load in direct-from-specimen AST, it was crucial to include two specimen dilutions to cover both ends of clinical microbial loads. We increased the dilution factor to 0.002×, resulting in 2 × 10^5^ CFU/mL for a 10^8^ CFU/mL sample, which is closer to the recommended 5 × 10^5^ CFU/mL. Additionally, starting sample volumes of 4-mL were previously used but proved to be difficult to obtain for remnant specimens, resulting in a smaller sample size^5^^,^^8^. To address this issue, we considered compared the effects of 4-mL and 2-mL starting volumes on low and high microbial loads.

### Specimen collection and transportation

All specimens were de-identified remnant aliquots from those collected by MCW for clinical diagnosis as part of their standard care. There were no human participants recruited. All samples were collected under a Non-Human Subject Research determination without consent (45 CFR 46 exemption 4) and in accordance with national regulations on the ethical involvement of human subjects. All specimens were collected in BD C&S Preservative tubes, packaged using the transportation pack, and shipped via UPS for next-day 8AM delivery^6^.

### Antibiotic stripwells and electrochemical sensor chips

Antibiotics (Cayman Chemical Company; Ann Arbor, MI, USA) of three different classes commonly prescribed for urinary tract infections were used: ciprofloxacin (CIP), ampicillin (AMP), sulfamethoxazole-trimethoprim (SMZ-TMP). Antibiotic stripwells were prepared using published protocols^9^. The first of 8 wells contained no antibiotic to function as the growth control (GC) and the remaining seven contained the following concentrations in order from wells 2 to 8: CIP—0.25, 1, 4, 0.0625, 0.25, 1, 4 µg/mL; AMP—8, 32, 128, 2, 8, 32, 128 µg/mL; SMZ-TMP (trimethoprim/sulfamethoxazole)—1/19, 2/38, 4/76, 0.5/9.5, 1/19, 2/38, 4/76 µg/mL. Electrochemical sensor chips were functionalized with probes to detect *Enterobacterales* and *Pseudomonas aeruginosa* using published methods^5^.

### Direct-from-urine AST assay

The direct-from-specimen AST was designed to be automated in a fully integrated, sample-to-result system ^5^. This study focuses on the benchtop evaluation of assay parameters to be incorporated into the robotic systems. Upon arrival, MCW specimens were incubated at 35 °C for 15 min prior to beginning the direct-from-specimen AST assay. Whenever specimen volume allowed, duplicates were tested with two operators to assess the reproducibility of results.

Urine samples of 2-mL volume were centrifuged for 15 min at 2719*g*. Supernatant was removed, leaving 0.5 mL, after which 3.5 mL of MH broth were added. The samples underwent a second round of centrifugation of the same conditions, after which 3.4 mL of supernatant were removed, leaving 0.6 mL of bacterial pellet. One-hundred microliters of this undiluted specimen (1×) was delivered to the first four wells of the antibiotic stripwell. The remaining 0.2 mL was diluted down to 0.002×, after which 100 µL were delivered to the last four wells; stripwells were incubated for 2 h at 37 °C. After the incubation, each well was lysed with 36 µL 1 M NaOH, followed by 5-min incubation at room temperature. Twenty-four microliters of 1 M HCl were added to each well, after which 15 µL of lysed sample were immediately delivered onto two corresponding sensors in one sensor chip. Sensor chips were incubated at 43 °C for 30 min, then washed with distilled water and dried with pressurized air. Ten microliters of horseradish peroxidase (HRP) were added to all 16 sensors and incubated for 5 min at room temperature. The sensor chip was washed and dried for a second time and 40 µL of 3,3',5,5'-tetramethylbenzidine (TMB) were delivered to all sensors. After a 30-s incubation at room temperature, the sensor chip was read with our potentiostat to obtain amperometric signal.

### Statistical analysis and susceptibility reporting

Data were analyzed using Analyse-it Ultimate Edition (Leeds, United Kingdom). The chi-square test was used to examine for associations between susceptibility and parameters extracted from the response curves of each dilution. Due to the lack of intermediate susceptibility from MCW specimens, this feasibility study consisted of only dichotomized resistance data (S vs. R); advanced statistical models would be needed for follow-up studies to analyze susceptibility prediction models in full-scale minimum inhibitory concentration distributions. The susceptibility reporting follows sequential rules to quantify the characteristics of the growth response curve against a spectrum of antimicrobial conditions; these characteristics are then matched to the unique signatures of susceptible and resistant strains. The reporting method places an emphasis on identifying resistant patterns in order to avoid very major errors in which resistant strains are reported susceptible. All antibiotics used the same algorithm and reporting parameters listed in Supplemental Tables S7–S9:

1. GC—The signal level from the growth control well in nanoampere (nA).
2. uLoad—Estimated microbial load based on the signal level of the GC well.
3. AccuDrop—This parameter indicates the accumulated drop in signal level from well 1 to well 8 and is the first round of reporting to identify high-confidence S results. If there is no significant change of signal levels across each specimen dilution, the sample is considered to be R and moves onto the next step; if there is a significant drop in signal levels and/or a decreasing trend in either specimen dilution, the sample is reported S.
4. AccuDrop%—This parameter is the percentage from dividing AccuDrop by the GC signal and is used to identify high-confidence R results. It is expected to have a low value for R strains due to insignificant growth inhibition across all antimicrobial conditions. However, AccuDrop% could be low for S strains of low microbial loads, indicating the need for the Plateau parameter. Plateau identifies uninhibited growth only across wells 5 and 6 (beginning of 0.002× specimen dilution) for the case in which the 1× response curve is saturated and the initial response of the 0.002× curve is critical. If Plateau is true, the sample is considered to be R.
5. Drop%—This parameter is the percentage of signal level decrease from the highest signal level to the Max.drop parameter value. Max.drop indicates the maximum growth inhibition and the signal reduction is calculated by subtracting the reporting signal of the following well from the current well. The location of the well where Max.drop occurs is recorded in the parameter, SW#MaxDrop. If SW#MaxDrop is recorded from 1 to 4 or 5 to 8, the most significant growth inhibition is in the 1× or 0.002× response curve, respectively. Drop% indicates the significance of the maximum growth inhibition within the specimen dilution where it occurs. S strains are expected to exhibit significant signal reduction and high Drop% at the bug-to-drug ratio (well) above the S-breakpoint bug-to-drug ratio determined by dividing 5 × 10^5^ CFU/mL by the S-breakpoint concentration for the respective antimicrobial. S strains with a low microbial load would have a low AccuDrop% but high Drop%.

The antimicrobial concentrations for SMZ-TMP followed a twofold dilution due to limitations in manufacturing, resulting in a more narrow spectrum compared to that of AMP and CIP, which utilized fourfold dilutions of antimicrobial solution. The change in the response curve is expected to be smaller for SMZ-TMP; the susceptibility reporting criteria for this antimicrobial was adjusted accordingly.

## Result

### Optimization studies with MCW remnant specimens

Conditions tested in the optimization study included 30% HRP, 1.5- and 4-h antimicrobial exposure times, and varying specimen dilutions. As shown in the Supplementary Figs. S1–S5 and Tables S1–S4, conditions including 1× specimen dilution and longer exposure times were generally more favorable for lower microbial loads closer to the limit of detection of 10^5^ CFU/mL. Conditions including diluted specimen and 100% HRP were preferred for higher microbial loads for outpatient settings. Detailed explanations of the signal resolution for low and high microbial loads are found in the Supplementary Information.

### Direct-from-specimen AST clinical feasibility study

#### Addressing the limited dynamic range of response curves

**Figure 1:**
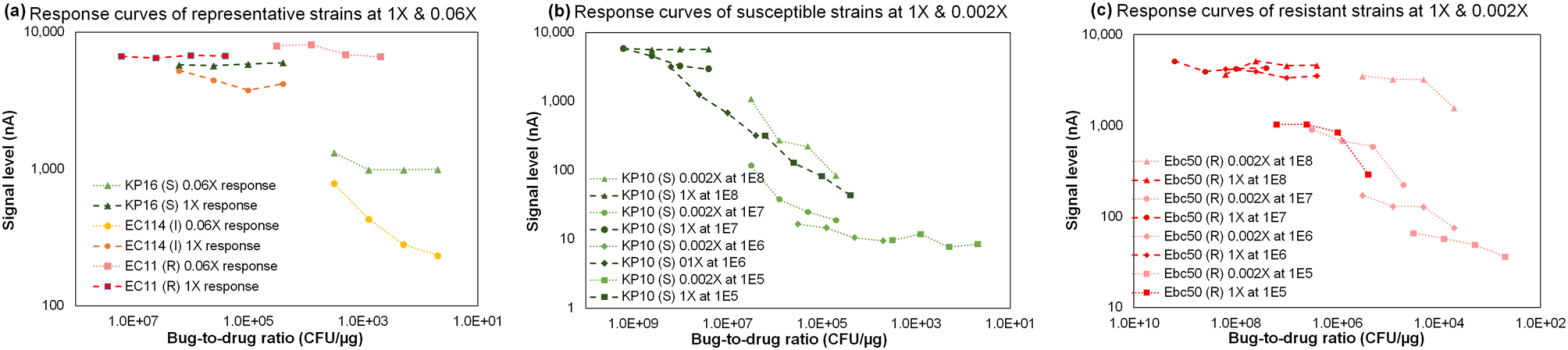
Specimen dilution configurations of (**a**) 1×/0.06× with representative S, I, and R strains; (**b**) 1×/0.002× with representative S strains, and (**c**) 1×/0.002× with representative R strains. Each error bar represents 2–4 data points.

To address the limited responses observed in the optimization studies, we changed the second specimen dilution from 0.06× to 0.002×, resulting in a wider range of bug-to-drug conditions. Specifically, as shown in Fig. 1a, in which we tested 10^8^ CFU/mL samples against gentamicin, there is no clear difference in susceptibility trend for the resistant and susceptible strains illustrated by the 1× (dashed lines) or 0.002× (dotted lines) response curves. Both strains exhibit a nearly flat curve at both specimen dilutions. After changing the second specimen dilution, as shown in Fig. 1b and c, there is a larger distinction between the susceptible and resistant strains. Specifically, the susceptible strain in Fig. 1b demonstrated a clearer decrease in signal across the 0.002× curve compared to the almost flat curve shown in Fig. 1a. The only significant drop in Fig. 1c is at the end of the 0.002× response curve (well# 7 to 8), which is below the equivalent bug-to-drug ratio R breakpoint at 5 × 10^5^ CFU/mL.

#### Addressing the variation in clinical sample volume

The optimization studies followed previously published studies by utilizing a 4-mL starting specimen volume. However, this larger sample volume presented limitations in the collection process, resulting in difficulty collecting sufficient volume and a smaller sample size. Therefore, we assessed the effects of a 2-mL starting volume on the signal resolution and resulting response curves. Results demonstrated that signal levels from the direct-AST assay did not change for higher microbial loads but did decrease for lower microbial loads. However, this decrease was minimal and was addressed by continuing to include the 1× specimen dilution.

**Figure 2:**
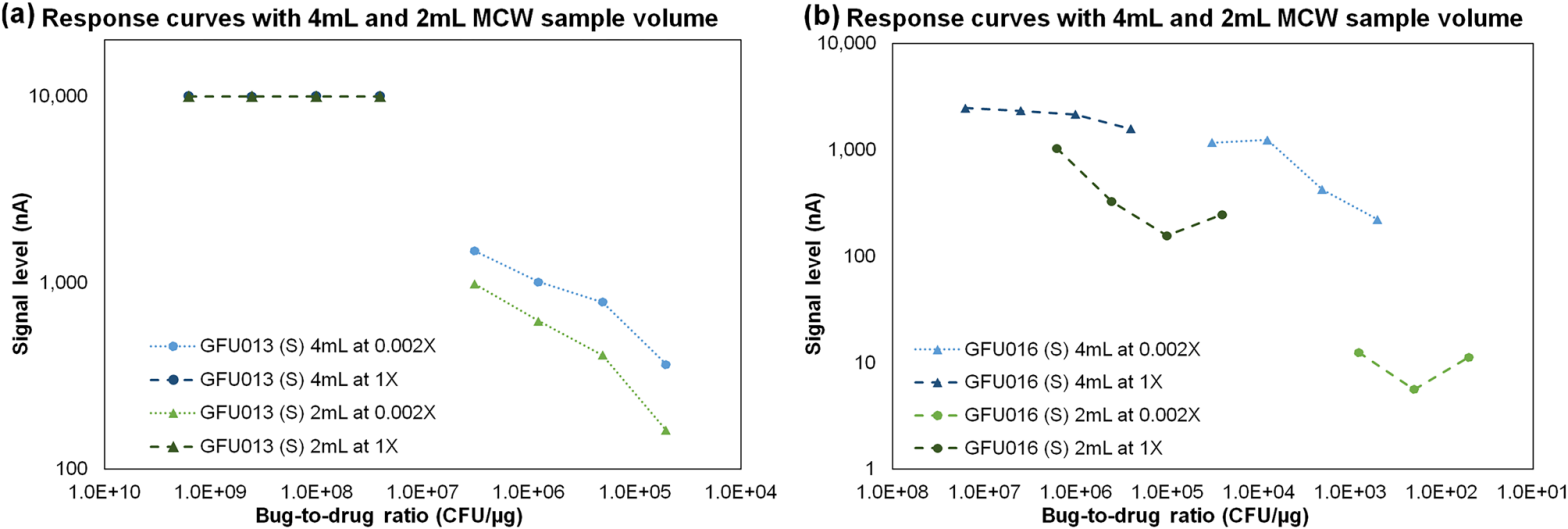
Representative results of MCW direct-from-specimen AST with different specimen volume. Conditions include 100% HRP, 3-h antibiotic exposure time, 1×/0.002× specimen dilution concentrations, comparison of 4-mL and 2-mL starting volumes. (**a**) Susceptible high microbial load specimen with starting volume of 4 mL and 2 mL, (**b**) susceptible low microbial load specimen with starting volume of 4 mL and 2 mL. Each error bar represents 2 data points.

Figure 2 displays partial results of the MCW direct-from-specimen AST clinical feasibility study, in which we applied all changes described in Supplementary Figs. S1–S5 to our direct-from-specimen AST. We changed four parameters: return to 100% HRP concentration instead of 30%, 3-h antibiotic exposure time rather than 4 h to shorten the assay as much as possible, 1×/0.002× specimen dilution instead of 1×/0.06×, and a 2 versus 4-mL starting volume. Figures 2a and b illustrate the growth response curves for two MCW specimens of a higher microbial load of 10^8^ CFU/mL and a lower microbial load possibly closer to the clinical cutoff of 1 × 10^3^ CFU/mL, respectively. Both strains were susceptible to ciprofloxacin and were reported correctly. It is apparent that the 100% enzyme concentration returned the 1× wells of the 1 × 10^8^ CFU/mL to saturation. The 3-h exposure time allowed lower concentrations to grow to a point of easier identification of susceptible trends, as observed in the 0.002× wells of the high-load sample in Fig. 2a and the 1× wells of the low-load sample in Fig. 2b. While the longer exposure time allowed us to more easily observe response curves for low and high concentration samples, we still observed signal level saturation for the 1× wells of high microbial loads.

Furthermore, the use of a 1× and 0.002× specimen dilution also contributed to the clear susceptible trend of the growth curve. In the comparison of the 4-mL and 2-mL samples in Fig. 2, the 1× wells of both the 4-mL and 2-mL 1 × 10^8^ CFU/mL samples remained saturated. However, the signal level of the 0.002× wells of the 1 × 10^8^ CFU/mL samples and the overall signal level of the 1 × 10^5^ CFU/mL samples exhibited an overall decrease. Despite this decrease, the susceptible trend was still apparent for these lower concentrations.

**Figure 3:**
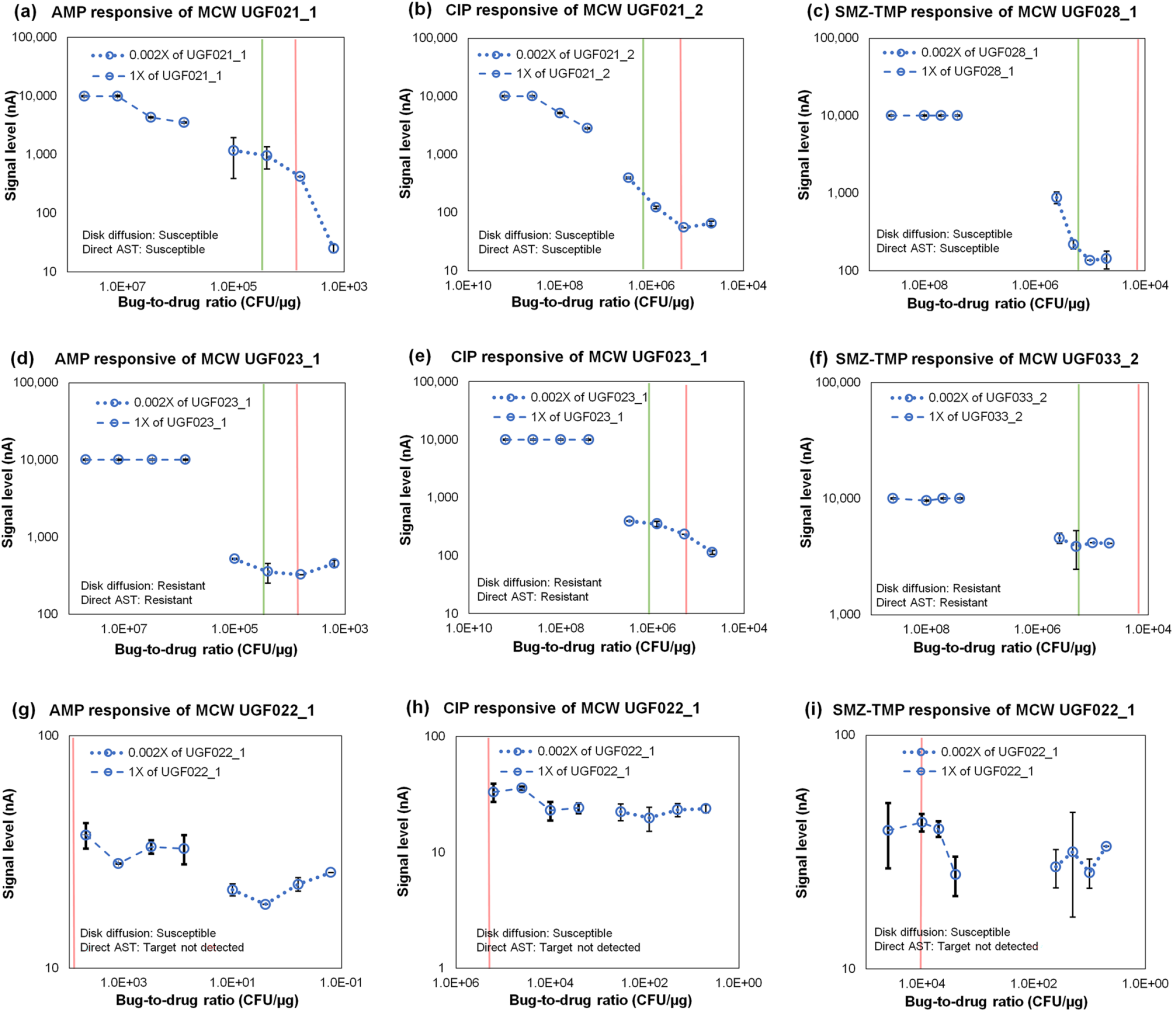
Representative results from MCW clinical feasibility study. (**a**) AMP susceptible, (**b**) CIP susceptible, (**c**) SMZ-TMP susceptible, (**d**) AMP resistant, (**e**) CIP resistant, (**f**) SMZ-TMP resistant, and Target not detected for UGF022_1 in (**g**) AMP, (**h**) CIP, and (**i**) SMZ-TMP AST. Each error bar represents 2 data points.

Figure 3 displays representative response curves from the direct-from specimen AST clinical feasibility in which we tested de-identified remnant specimens from MCW with the optimized protocol; test results are summarized in Table 1. Further information on individual reporting and testing conditions can be found in Supplementary Table S5; reporting parameter values are listed in Supplementary Tables S7–S9. Response curves for all other specimens are displayed in Supplementary Figs. S6–S8. The major difference between this clinical feasibility study and our previously published studies is use of non-screened clinical specimens. Specifically, MCW shipped prospectively collected clinical specimens without waiting for pathogen ID results to reduce the time from specimen collection to direct-from-specimen AST result reporting. Therefore, a total of 36 out of 97 total specimens were reported as “Target not detected” due to either (1) target pathogen at a microbial load lower than the clinical cutoff, (2) pathogens not on the target ID panel, or (3) mixed flora of both (1) and (2). Six specimens were reported “Invalid” due to the failure of internal controls including (1) signal separation between the two specimen dilutions to avoid calling susceptibility with saturated or extreme signal levels, and (2) abnormal curve characteristics caused by reading errors.

**Table 1:** Direct-from-specimen AST clinical feasibility study – 1×/0.002× configuration. The categorical agreement for ampicillin is calculated not including the unauthenticated results for UGF039_1 and UGF039_2.

Direct-from-urine AST results	Reference disk diffusion results			Evaluation	
	S	I	R	Categorical agreement	
**Antibiotic: Ampicillin**					
S	10	0	0	14/16	87.5%
I	0	0	0		
R	2	0	4		
**Antibiotic: Ciprofloxacin**					
S	21	0	0	23/23	100%
I	0	0	0		
R	0	0	2		
**Antibiotic: Sulfamethoxazole-trimethoprim**					
S	10	0	0	14/14	100%
I	0	0	0		
R	0	0	4		

Representative response curves of clinical specimens with confirmed susceptibility in Fig. 3a–c exhibit quantifiable inhibited characteristics of at least one significant signal drop in either or both of the specimen dilution curves. A significant drop in signal level can be observed in a typical responsive curve, either in the 1× dilution (wells 1–4) or 0.002× dilution (wells 5–8), from a susceptible strain. For high microbial loads, the 1× response curve may be saturated and characterized as a plateau as shown in Fig. 3c, while the 0.002× response curve exhibits a significant signal drop. For medium microbial loads, there may not be a significant signal drop; however, a consistent decreasing trend can be observed from 1× to 0.002× response curve as demonstrated in Fig. 3b. Distinctly different characteristics of representative response curves of resistant clinical specimens plotted in the log scale in Fig. 3d, e, and f exhibit typical characteristics of uninhibited growth such as saturated growth of the 1× curve in Fig. 3d and no significant signal drop in the 0.002× curve. Specimens reported as “Target not detected” can be identified by two characteristics: (1) no difference in the signal level between the 1× and 0.002× curves and (2) all signal levels < 150 nA as demonstrated in Fig. 3g, h, and i.

**Figure 4:**
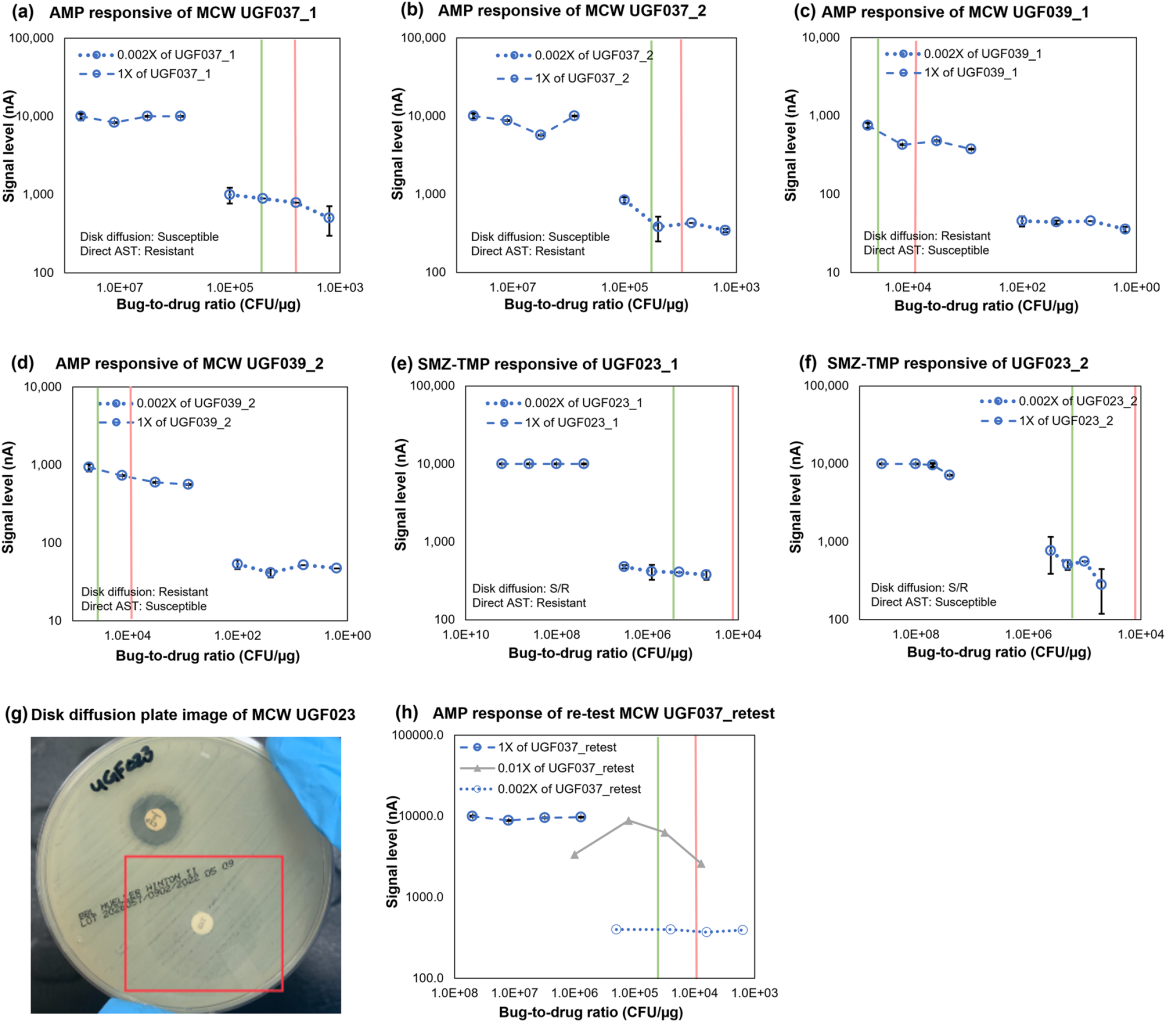
Discrepancies between direct-from-specimen AST and disk diffusion from MCW clinical feasibility studies. (**a**–**f**) Response curves of discrepancies, (**g**) disk diffusion result for UGF023, (**h**) re-test of UGF037 against ampicillin including extra 0.01× specimen dilution. Each error bar represents 2 data points.

Figure 4 illustrates the discrepancies observed during the clinical feasibility study; re-test results for these strains are available in Supplementary Table S6. There were two confirmed major errors shown in Fig. 4a and b for UGF037_1 and UGF037_2, in which susceptible samples were reported resistant. UGF037 was retested with the same conditions but continued to exhibit a resistant response curve with a susceptible disk diffusion result. After the addition of a 0.01× specimen dilution as shown in Fig. 4h, we were able to observe a susceptible response for UGF037, suggesting that neither the 1× (designed for low microbial loads at around the LoD) nor the 0.002× (designed for high microbial loads ≥ 1 × 10^8^ CFU/mL) specimen dilution contained the bug-to-drug ratio range that carried the growth inhibition response. There were also two confirmed very major errors shown in Fig. 4c and d, in which resistant samples were reported susceptible; both results were unable to be reproduced in the re-tests to identify the failure modes and the original specimen was not available for further investigation. Additionally, we exhibited a potential polymicrobial response against SMZ-TMP for UGF023_1 and UGF023_2. Figure 4e and f display contradicting direct-from-specimen AST results showing both susceptible and resistant responses; Fig. 4g displays the disk diffusion image for UGF023 with both susceptible and resistant growth. The resistant response is indicated by the flat response curves in Fig. 4e and the microbial growth inside the zone of inhibition zone in Fig. 4g, while the susceptible response is indicated by the decreasing trend in the 0.002× curve shown in Fig. 4f and the growth outsize of the zone of inhibition in Fig. 4g.

## Discussion

There has been a great deal of effort to develop direct-from-specimen AST systems or rapid AST assays, but major limitations have been a lack of susceptibility agreement with CLSI reference methods and the need for clinical isolates derived in the clinical microbiology laboratory^10–12^. Previously, we presented a method to quantify the species- or group-specific 16S rRNA content of viable target pathogens in unprocessed urine; we developed a method to interpret the antimicrobial effect by analyzing the microbial responses at two dilutions of specimen against identical antibiotic conditions^5,8^. The inoculum concentration (standard 5 × 10^5^ CFU/mL vs. unknown), pathogen population (monomicrobial vs. polymicrobial), and growth rate (log phase vs. stationary phase) are well controlled through the standardized inoculation using individual colonies from a sub-culture to ensure the microbiological response from susceptible to resistant strains can be fully covered by the dynamic range of the detection technology. However, in direct-from-specimen AST methods, such conditions are not as well-controlled. In the presented study, we highlighted our approaches to overcoming the most challenging barrier in performing direct-from-specimen AST: unknown microbial load of specimen. Despite the additional challenge of unknown pathogen population, the unknown microbial load in unprocessed clinical specimen is the greatest deviation from the current standard AST procedure. The goal of the present direct-from-specimen AST is to rule out ineffective antibiotics; therefore, the priority lies in high categorical agreement with reference methods and minimum very major errors (VME).

There are different factors that must be taken into consideration when performing direct-from-specimen AST, as varying conditions may significantly alter the detection capabilities of this method. The target microbial load range dictates the conditions of the direct-from-specimen AST. In terms of outpatient settings, positive ID results usually indicate ≥ 1 × 10^3^ CFU/mL. However, there still exist great differences in microbiological behavior at either end of that range as observed in the presented studies. For loads closer to the threshold of 1 × 10^3^ CFU/mL, it is necessary to include the undiluted 1× specimen dilution and sufficient exposure time to allow for reliable detection of bacteria and distinction between susceptible and resistant strains. For loads on the higher end of the spectrum at ≥ 1 × 10^8^ CFU/mL, it is essential to include diluted specimen of a larger dilution factor and 100% enzyme concentration. Therefore, for direct-from-specimen AST performed in outpatient settings with a goal of testing above the clinical threshold, such conditions are required to accommodate both ends of the spectrum. In the special cases of inpatient specimens obtained from catheters, for example, the extremely low microbial loads of ≤ 10 CFU/mL would require a different set of direct-from-specimen AST conditions, such as larger starting volume and longer exposure times, which further emphasize the notion that the conditions of direct-from-specimen AST are influenced by the microbiological characteristics expected from different standard of care procedures and their corresponding sources of specimens.

In the optimization studies, we attempted to address the major errors observed for high microbial load specimens by adjusting the signal levels through varying enzyme conditions. However, this approach did not resolve the suppressed response curves caused by signal saturation and simply decreased the detection sensitivity. Therefore, a lower specimen dilution of 0.002× from 0.06× was adopted to ensure that at least one of the four bug-to-drug ratios of the lower specimen dilution would be equivalent or close to the standard inoculum of 5 × 10^5^ CFU/mL used in CLSI reference methods. If the original microbial load in the unprocessed specimen were 1 × 10^8^ CFU/mL, the 0.06× specimen dilution would be equivalent to 6 × 10^6^, which is still higher than the standard inoculum of 5 × 10^5^ CFU/mL; however, a specimen dilution of 0.002×, which is equivalent to 2 × 10^5^ CFU/mL and is closer to the standard inoculum concentration. Susceptibility breakpoints are established with fixed bug-to-drug ratios with a bug concentration of 5 × 10^5^ CFU/mL. When the microbial load is much higher or lower than 5 × 10^5^ CFU/mL, the response curves become suppressed by the signal reporting range. The priority is to avoid VME; therefore, the signal must be at the highest possible levels in order to report uninhibited growth for the resistant strains. The next priority is to avoid major error (ME), in which the S strain is reported R; therefore, at least one of the specimen dilutions must be equivalent or near the standardized inoculum concentration. The response curves in Supplementary Fig. S3 and S4 demonstrated that the 0.002× specimen dilution can effectively reduce the rate of ME.

Just as empirical antibiotic therapy is determined by ruling out the most likely ineffective antibiotics based on the resistant trends from the local antibiogram, the goal of the patient-specific direct-from-specimen AST is to rule out ineffective antibiotics and rule in antibiotics that inhibit bacterial growth. To do so, we take a qualitative approach by prioritizing the trend of the resulting growth curve over the actual signal level generated from each antibiotic condition. The direct-from-specimen AST susceptibility reporting is based on a three-tier analysis as detailed in the Methods. The purpose of the described parameters in Tables S7–S9 is to quantify and standardize the distinct susceptibility characteristics in order to match manual predictions based on the response curves. The first two rounds of analysis are used to report susceptibility with high-confidence response characteristics for S and R strains based on the parameters extracted from both specimen dilution curves. Figure 3a, b, and c were reported S with high confidence with AccuDrop = 7624.7 for UGF021_1 against AMP, AccuDrop = 7505 for UGF021_2 against CIP, and Drop% + AccuDrop% = 82% for UGF028_1 against SMZ_TMP. Figure 3d, e, and f were called R with high confidence with AccuDrop% = 1% for UGF023_1 against AMP, AccuDrop% = 3% for UGF023_1 against CIP, and AccuDrop% = 4.5% for UGF033_2 against SMZ-TMP. Figure 3g, h, and i were reported “Target not detected” with all signal levels under 150 nA. The susceptibility parameters for all MCW specimens in the clinical feasibility study are detailed in Tables S7–S9. After AccuDrop reports high-confidence S and AccuDrop% reports high-confidence R samples, the remaining response curves are considered to be either borderline S and R or contain mixed susceptibility characteristics. The AST reporting for these samples is determined by the overall susceptibility levels calculated from all parameters. The estimated microbial load is determined by the resulting amperometric signal level from the GC well. The correlation between the signal output and estimated microbial load (CFU/mL) using the plate count of the CLSI density check method was previously published and used as a proficiency test for the medical technologists participating in our clinical testing^13^.This estimated load is then used as a criterion to assess the change in signal levels (or microbial growth) above the standard inoculum of 5 × 10^5^ CFU/mL used in CLSI reference methods to ensure compatibility with laboratory standards used in clinical settings.

The response curves of the direct-from-specimen AST relative to the S (green line) and R (red line) breakpoints at the standardized 5 × 10^5^ CFU/mL inoculum used in reference methods are overlaid in Fig. 4 for comparison. UGF037_1 and UGF037_2 exhibit response curves with borderline R and S characteristics as shown in Fig. 4a and b. Both were called R due to several parameters reporting values too close to the values from R strains; the re-test confirms no significant growth inhibition, indicated by the low signal levels and flat characteristic of the 0.002× responsive curves. The S-breakpoint represented by the green line is located at the start of the 0.002× responsive curve and the disk diffusion result exhibits medium susceptibility. These two findings indicate that the most significant growth inhibition (signal drop) may have occurred within the gap between the 1× and 0.002× response curves, resulting in a flatter line in the 0.002× curve due to bug-to-drug ratios far below the S-breakpoint. Previous versions of the AST stripwell covered the entire spectrum with overlapping bug-to-drug ratios between the two response curves using two stripwells and sensor chips per sample; however, we decided to cover only the most common microbial loads, which are right above the LoD and above 1 × 10^7^ CFU/mL, with just one stripwell and one sensor chip to reduce the assay cost and complexity. After adding a 0.01× specimen dilution in Fig. 4h, we were able to observe the susceptible response, thus confirming our hypothesis that neither the 1× nor the 0.002× contained the drop. Although the 0.01× and 0.002× curves slightly overlap and ideally would demonstrate the same response curve for the ratios in which they overlap, it is possible that the 0.002× specimen dilution resulted in a bacterial concentration lower than what was expected, thus resulting in a flat curve. Both UGF039_1 and UGF039_2 were reported S in Fig. 4c and d; however, the re-test and disk diffusion both reported R. Neither of these results were able to be reproduced. The re-test of the UGF039 agreed with the disk diffusion, and the VME failure mode could not be investigated due to non-reproducibility. We are not able to authenticate this sample with reproducible results; therefore, the CA was calculated excluding these two samples as noted in Table 1. Samples UGF023_1 and UGF023_2 for SMZ-TMP were reported “Invalid” due to our inability to authenticate this sample with the disk diffusion result shown in Fig. 4e, f, and g. The disk diffusion result exhibited mixed flora of both R and S trains; additionally, the direct-from-specimen AST also reported both R and S, resulting in no direct comparison, which we intended to accomplish with this study.

As demonstrated, this approach of microbial growth inhibition response curves to antibiotic exposure conditions across microbial loads ranging from overgrowth, which is common in outpatient settings, to an estimated clinical cutoff of 1 × 10^3^ CFU/mL, which is more frequent in inpatient settings, can provide a dynamic and rapid method for estimating antimicrobial efficacy in a much shorter timeframe than the endpoint minimum inhibitory concentration (MIC) method used in conventional AST. The polymicrobial vs. monomicrobial challenge can be addressed in future studies with the addition of species-specific oligonucleotide probes on additional sensors to quantify the growth of each present organism or group after the antimicrobial exposure.

## Supplementary Information

Supplementary Information.

## Data Availability

The datasets used and/or analyzed during the current study available from the corresponding author on reasonable request.
