# Supplementary material for: Real world clinical feasibility of direct-from-specimen antimicrobial susceptibility testing of clinical specimens with unknown microbial load or susceptibility
Source: Sci Rep. 2022 Nov 2;12:18525. doi: 10.1038/s41598-022-21970-2 (PMC9630444; doi:10.1038/s41598-022-21970-2)
Supplement: Supplementary file 1 — Supplementary Information. [file 41598_2022_21970_MOESM1_ESM.docx]

**Title:** Real world clinical feasibility of direct-from-specimen antimicrobial susceptibility testing of clinical specimens with unknown microbial load or susceptibility

Jade Chen^1^, Eduardo Navarro^1^, Brian Mesich^2^, Derek Gerstbrein^2^, Amorina Cruz^2^, Matthew L. Faron^2^, Vincent Gau^1*^

^1^ GeneFluidics, Los Angeles, California, USA

^2^ The Medical College of Wisconsin, Milwaukee, Wisconsin, USA

*Corresponding author: vgau@genefluidics.com

**SUPPLEMENTAL MATERIALS**


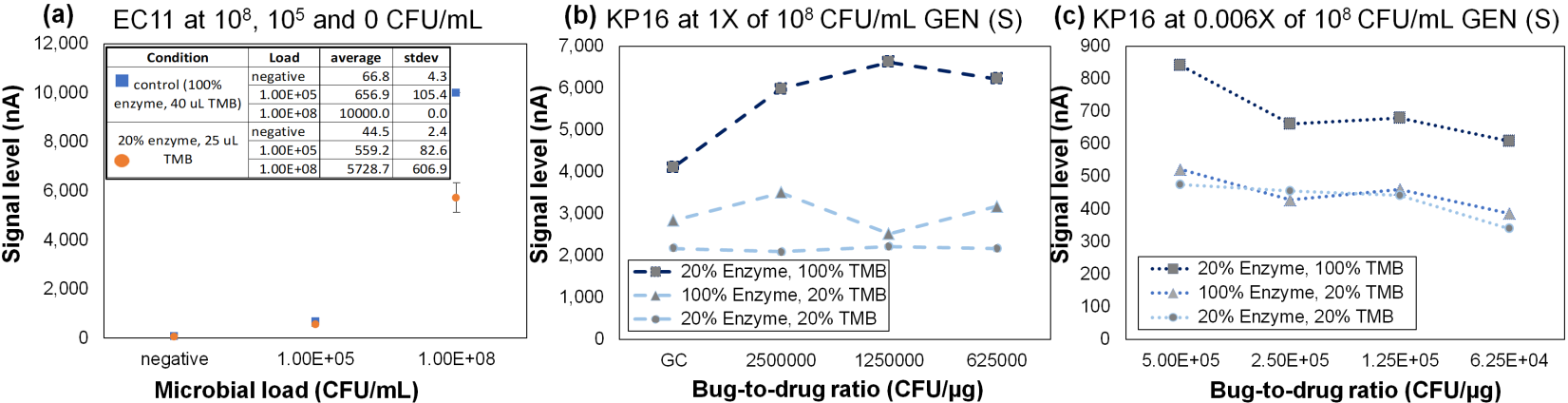


**Figure S1.** Varying HRP and TMB concentrations and their effects on signal level saturation. (a) ID assay results testing 10^5^ and 10^8^ CFU/mL samples, (b) additional HRP and TMB conditions tested with the direct-from-specimen AST using a 10^8^ CFU/mL sample. Each error bar represents 2-7 data points. This experiment addressed the skewed response curves from specimens with high microbial loads (≥10^8^ CFU/mL). Previous publications revealed signal level saturation of wells containing 1X inoculum for samples of high microbial load ≥10^8^ CFU/mL, which is typical of outpatient urine cultures. In a pilot test prior to the MCW clinical feasibility study, we experienced difficulty in assessing the overall susceptibility trend of the growth curve due to the 1X signal level being out of range (≥10,000 nA, the signal reporting limit). Here, we demonstrate that decreasing the HRP or TMB concentration can attenuate the corresponding signal level of the 1X wells and therefore avoid signal saturation. As demonstrated in Fig. S1a, we successfully decreased the signal of the 10^8^ CFU/mL sample without significantly changing the signal level of the 10^5^ CFU/mL. We then tested more conditions in the direct-from-specimen AST with a 10^8^ CFU/mL sample; Fig. S1b illustrates the same results of signal level reduction for the 1X wells containing 10^8^ CFU/mL and no dramatic changes for the 0.06X wells containing 6×10^6^ CFU/mL. Although all three conditions were able to eliminate the signal saturation of 1X wells, only the first condition of 20% HRP and 100% TMB avoided a decrease in signal level for the 0.06X wells. Still, the resulting 0.06X signal level from condition A was lower than desired; therefore, we continued with a slightly higher 30% HRP concentration in the subsequent MCW optimization studies.


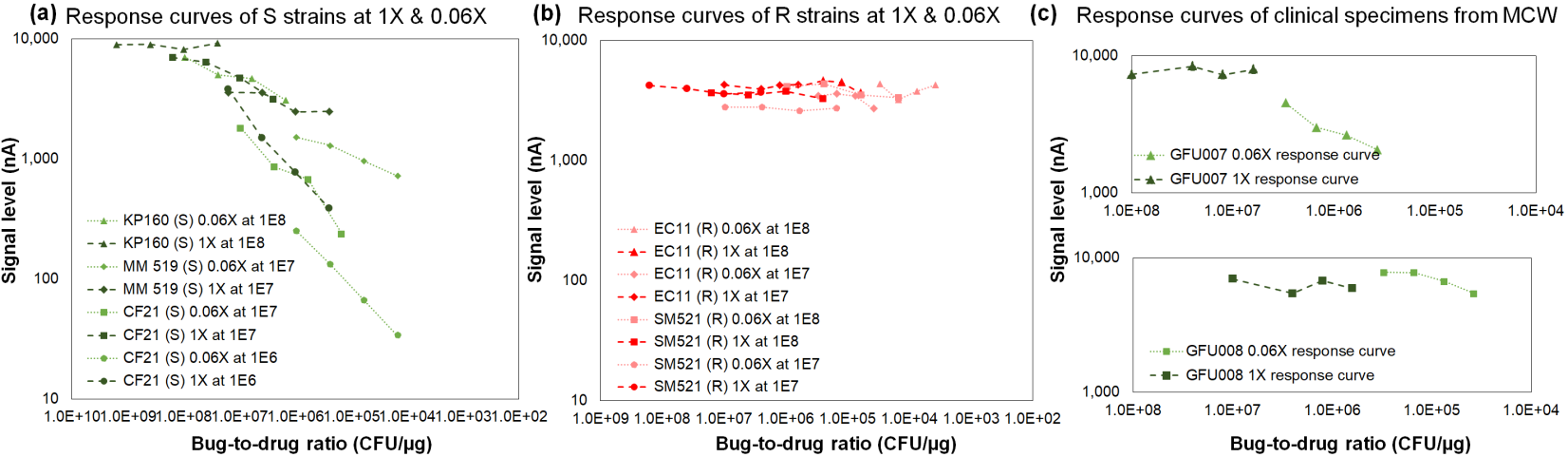


**Figure S2.** Representative results from MCW optimization enzyme study using 30% HRP. (a) Growth response curve of 10^8^ CFU/mL strains susceptible to ciprofloxacin using 100% HRP, (b) growth response curve of 10^8^ CFU/mL strains resistant to ciprofloxacin using 100% HRP, (c) Representative result of MCW clinical specimen GFU007 and GFU008 susceptible to ciprofloxacin using 30% HRP. The curves in Fig. S2c resembled that of a typical resistant strain, shown in Fig. S2b rather than a susceptible strain, shown in Fig. S2a. This trend is likely due to the reduction of HRP, which affected only the 1X response and not the 0.06X response, lowering the 1X curve to meet the 0.06X curve and causing a resemblance to a resistant strain. Additionally, the reduced HRP concentration created a new signal cap ranging from 4,000 to 6,000 nA instead of the original 10,000 nA limit.

**Table S1.** Summary of MCW optimization HRP study in Figure S2. All samples were tested in duplicate with two different operators.

| **MCW specimen** | **Standard of care microbial load** | **Standard of care pathogen ID** | **Gentamicin disk diffusion measurement (mm)** | **Gentamicin reference susceptibility** | **Gentamicin direct-from-specimen AST reported susceptibility** |
| --- | --- | --- | --- | --- | --- |
| GFU001­_1 | >10^5^ CFU/mL | *E. coli*, other | 20 | Susceptible | Resistant |
| GFU001­_2 |  |  |  |  | Susceptible |
| GFU002_1 | >10^5^ CFU/mL | *P. mirabilis*, gram-negative rods, other | 21 | Susceptible | Intermediate |
| GFU002_2 |  |  |  |  | Resistant |
| GFU003_1 | >10^5^ CFU/mL | *E. coli* | 20 | Susceptible | Intermediate |
| GFU003_2 |  |  |  |  | Susceptible |
| GFU004_1 | >10^5^ CFU/mL | *K. pneumoniae*, other | 22 | Susceptible | Resistant |
| GFU004_2 |  |  |  |  | Resistant |
| GFU005_1 | >10^5^ CFU/mL | *K. oxytoca*, other | 21 | Susceptible | Resistant |
| GFU005_2 |  |  |  |  | Susceptible |
| GFU006_1 | >10^5^ CFU/mL | *P. mirabilis*, other | 22 | Susceptible | Resistant |
| GFU006_2 |  |  |  |  | Intermediate |
| GFU007_1 | Not available | Multiple bacteria isolated | 21 | Susceptible | Resistant |
| GFU007_2 |  |  |  |  | Resistant |
| GFU008_1 | >10^5^ CFU/mL | *E. coli*, other | 21 | Susceptible | Resistant |
| GFU008_2 |  |  |  |  | Resistant |
| GFU009_1 | >10^5^ CFU/mL | *E. coli* | 23 | Susceptible | Intermediate |
| GFU009_2 |  |  |  |  | Intermediate |
| GFU010_1 | >10^5^ CFU/mL | *E. coli*, other | 22 | Susceptible | Intermediate |
| GFU010_2 |  |  |  |  | Resistant |
| Categorical Agreement | | | | | 3/20 = 15% |
| Minor Errors | | | | | 6/20 = 30% |
| Major Errors | | | | | 11/20 = 55% |
| Very Major Errors | | | | | 0/20 = 0% |


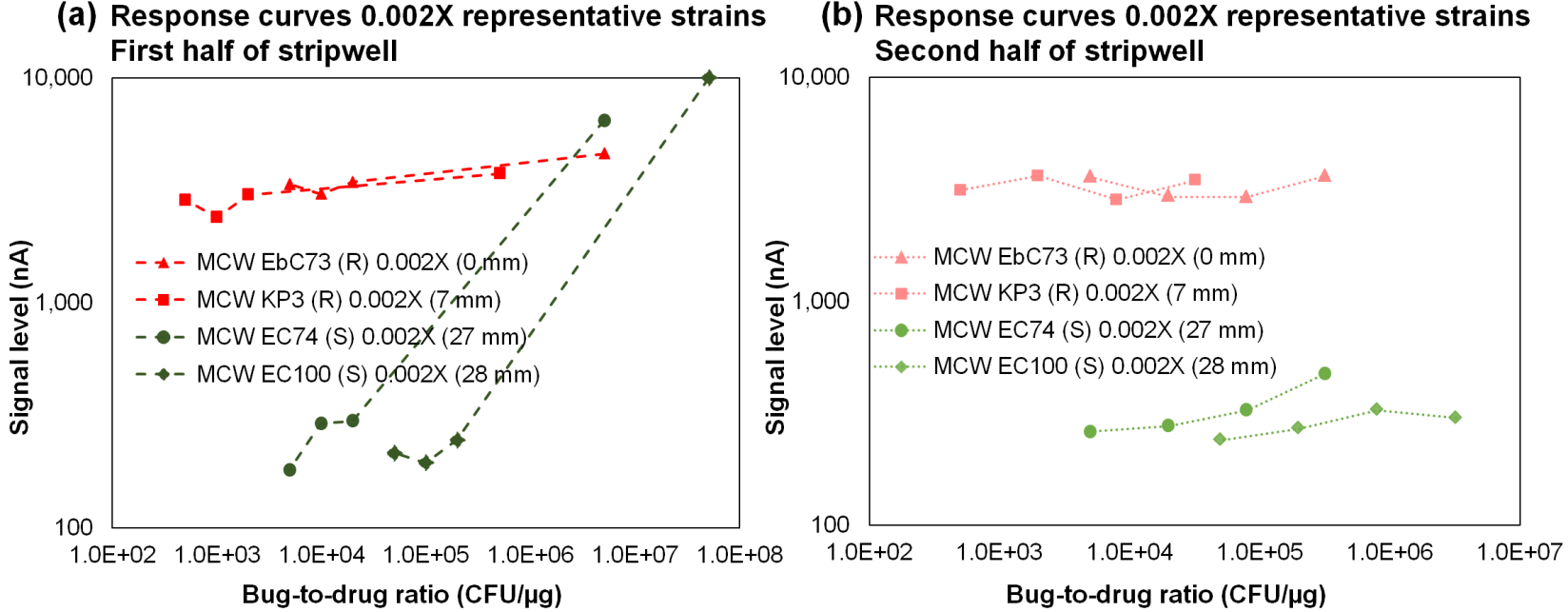


**Figure S3.** In-house high microbial load study using only 0.002X inoculum to address the throughput and cost of goods sold (COGS). (a) Strain susceptible to SXT, (b) Strain resistant to SXT. After in-house and optimization testing of specimens with high microbial loads, we observed continuous signal saturation for 1X wells, leading to the removal of 1X inoculum in the MCW optimization high microbial load study. Preliminary in-house testing showed sufficient growth and clear susceptibility trends using duplicate 0.002X inoculum rather than the 1X/0.002X configuration. The assay throughput and COGS would be significantly improved if the use of 1X inoculum were omitted. Figure S3 demonstrates the clear difference between susceptible and resistant curves for sulfamethoxazole-trimethoprim, with a substantial decrease in signal indicative of growth inhibition for the susceptible strains.

**Table S2.** Summary of in-house high microbial load study evaluating the use of only 0.002X inoculum against sulfamethoxazole-trimethoprim (SXT). Bacteria isolates used to contrive samples for in-house evaluations were obtained from the CDC AR Bank and de-identified remnant isolates from MCW. Isolates were stored as glycerol stocks at -20℃ and sub-cultured on tryptic soy agar plates with 5% sheep’s blood for contriving. Mueller-Hinton II (MH) broth was used as culture media for the AST incubation.

| Sample Number | Source | Organism | Strain Number | Reference susceptibility | Direct-from-specimen AST report |
| --- | --- | --- | --- | --- | --- |
| 1 | CDC | *Klebsiella aerogenes* | 18 | Susceptible | Susceptible |
| 2 | CDC | *Escherichia coli* | 19 | Susceptible | Susceptible |
| 3 | CDC | *Klebsiella pneumoniae* | 3 | Resistant | Resistant |
| 4 | MCW | *Klebsiella oxytoca* | 113 | Resistant | Resistant |
| 5 | MCW | *Klebsiella pneumoniae* | 3 | Resistant | Resistant |
| 6 | MCW | *Escherichia coli* | 74 | Susceptible | Susceptible |
| 7 | MCW | *Escherichia coli* | 100 | Susceptible | Susceptible |
| 8 | MCW | *Enterobacter cloacae* | 73 | Resistant | Resistant |
| 9 | MCW | *Escherichia coli* | 4 | Resistant | Resistant |
| 10 | MCW | *Klebsiella oxytoca* | 113 | Resistant | Resistant |
| 11 | CDC | *Klebsiella pneumoniae* | 3 | Resistant | Resistant |
| 12 | MCW | *Citrobacter freundii* | 116 | Susceptible | Susceptible |
| Categorical Agreement | | | | | 12/12 = 100% |
| Minor Error | | | | | 0/12 = 0% |
| Major Error | | | | | 0/12 = 0% |
| Very Major Errors | | | | | 0/12 = 0% |


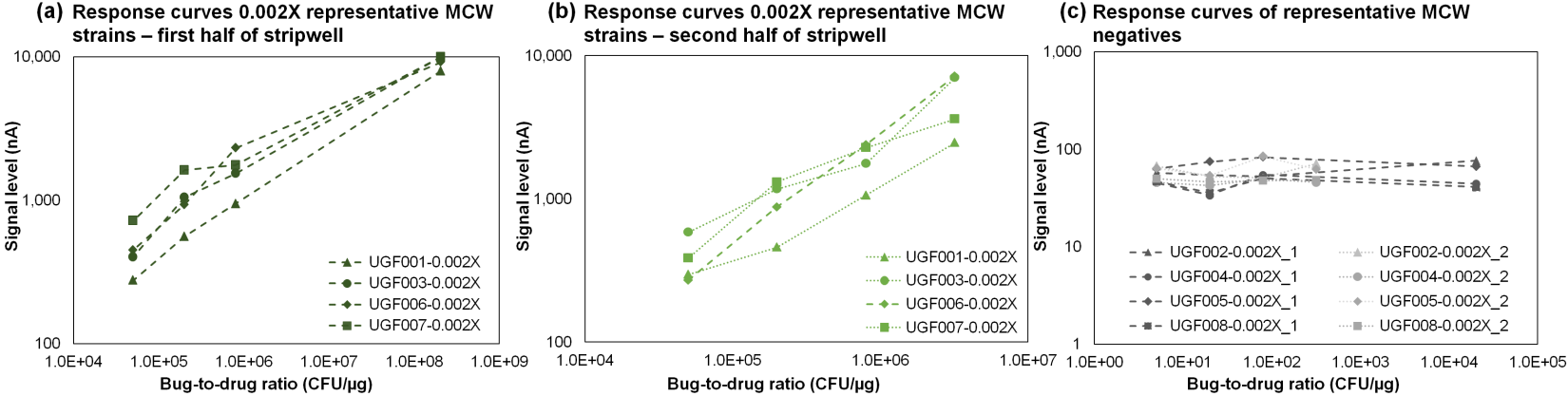


**Figure S4.** Representative results from MCW optimization high microbial load study. (a-b) susceptible response curves of susceptible strains against CIP, (c) response curves of samples reported “Target not detected”. Moving forward to the MCW optimization high microbial load study using the duplicate 0.002X inoculum, we encountered 70 reports of “Target not detected” (25 ampicillin, 22 ciprofloxacin, 23 sulfamethoxazole-trimethoprim) out of 108 total tests (36 ampicillin, 36 ciprofloxacin, 36 sulfamethoxazole-trimethoprim). Twenty-four of the seventy were due to blank samples containing a reference report of “No Growth”. Eighteen of the seventy were due to the specimens containing Gram-positive pathogens, which are not included in our target organism panel and therefore not detected by our sensors. Twenty-five of the seventy were due to specimens containing target pathogens at a concentration below the limit of detection and clinical threshold of 10^5^ CFU/mL. Three of the seventy were positive samples with a report of “>10^5^ CFU/mL”; these samples were likely closer to the 10^5^ CFU/mL end of the microbial load spectrum we had anticipated. Assuming these samples were at 10^5^ CFU/mL, the 0.002X inoculum concentration would have been 200 CFU/mL, which is not detectable with the conditions we established from other studies. Samples containing higher concentrations exhibited clear susceptibility trends. Based on these results, the 1X inoculum may only be omitted when the microbial loads are expected to be high, such as in urine cultures from outpatient settings.

**Table S3.** MCW optimization high microbial load study: testing parameters and reporting.

| **Ship date** | **MCW specimen** | **Standard of care microbial load (CFU/mL)** | **Standard of care pathogen ID** | **Start volume (mL)** | **Exposure time** | **AMP disk diffusion** | **AMP direct-from-specimen AST** | **CIP disk diffusion** | **CIP direct-from-specimen AST** | **SXT disk diffusion** | **SXT direct-from-specimen AST** |
| --- | --- | --- | --- | --- | --- | --- | --- | --- | --- | --- | --- |
| 03/03 | UGF001_1 | >10^5^ | Multiple organisms | 2 | 2 | 10 (R) | R | 30 (S) | S | 24 (S) | S |
|  | UGF001_2 |  |  | 2 | 2 |  | R |  | S |  | S |
| 03/03 | UGF002_1 | 5.5×10^4^ | *E. coli*, other | 2 | 2 | 0 (R) | Target not detected | 22 (I) | Target not detected | 27 (S) | Target not detected |
|  | UGF002_2 |  |  | 2 | 2 |  | Target not detected |  | Target not detected |  | Target not detected |
| 03/03 | UGF003_1 | >10^5^ | *E. coli*, other | 2 | 2 | 17 (S) | R | 28 (S) | S | 26 (S) | S |
|  | UGF003_2 |  |  | 2 | 2 |  | S |  | S |  | S |
| 03/03 | UGF004_1 | 2×10^3^ | Gram-positive | 2 | 2 | 22 (S) | Target not detected | 16 (R) | Target not detected | 27 (S) | Target not detected |
|  | UGF004_2 |  |  | 2 | 2 |  | Target not detected |  | Target not detected |  | Target not detected |
| 03/03 | UGF005_1 | No growth | N/A | 2 | 2 | N/A | Target not detected | N/A | Target not detected | N/A | Target not detected |
|  | UGF005_2 |  |  | 2 | 2 |  | Target not detected |  | Target not detected |  | Target not detected |
| 03/03 | UGF006_1 | >10^5^ | *P. mirabilis*, *E. coli, gram-negative rods,* other | 2 | 2 | 26 (S) | R | 35 (S) | S | 26 (S) | S |
|  | UGF006_2 |  |  | 2 | 2 |  | R |  | S |  | S |
| 03/03 | UGF007_1 | >10^5^ | *K. pneumoniae* | 2 | 2 | 10 (R) | R | 26 (S) | S | 25 (S) | S |
|  | UGF007_2 |  |  | 2 | 2 |  | S |  | S |  | S |
| 03/03 | UGF008_1 | 3×10^3^ | Gram-negative rods, other | 2 | 2 | 24 (S) | Target not detected | 29 (S) | Target not detected | 0 (R) | Target not detected |
|  | UGF008_2 |  |  | 2 | 2 |  | Target not detected |  | Target not detected |  | Target not detected |
| 03/03 | UGF009_1 | 4×10^4^ | *E. coli* | 2 | 2 | 18 (S) | Target not detected | 29 (S) | S | 27 (S) | Target not detected |
|  | UGF009_2 |  |  | 2 | 2 |  | Target not detected |  | Target not detected |  | Target not detected |
| 03/03 | UGF010_1 | No growth | N/A | 2 | 2 | N/A | Target not detected | N/A | Target not detected | N/A | Target not detected |
|  | UGF010_2 |  |  | 2 | 2 |  | Target not detected |  | Target not detected |  | Target not detected |
| 03/03 | UGF011_1 | 9×10^3^ | Gram-negative rods, other | 2 | 2 | 19 (S) | Target not detected | 30 (S) | Target not detected | 26 (S) | Target not detected |
|  | UGF011_2 |  |  | 2 | 2 |  | Target not detected |  | S |  | Target not detected |
| 03/03 | UGF012_1 | No growth | N/A | 2 | 2 | N/A | Target not detected | N/A | Target not detected | N/A | Target not detected |
|  | UGF012_2 |  |  | 2 | 2 |  | Target not detected |  | Target not detected |  | Target not detected |
| 03/03 | UGF013_1 | 1×10^3^ | Gram-positive | 2 | 2 | 21 (S) | Target not detected | 26 (S) | Target not detected | 26 (S) | Target not detected |
|  | UGF013_2 |  |  | 2 | 2 |  | Target not detected |  | Target not detected |  | Target not detected |
| 03/03 | UGF014 | 3×10^3^ | Gram-negative rods, other | 2 | 2 | 0 (R) | Target not detected | 33 (S) | Target not detected | 23 (S) | Target not detected |
| 03/03 | UGF015 | 1.8×10^4^ | Yeast | 2 | 2 | 0 (R) | Target not detected | 0 (R) | Target not detected | 0 (R) | Target not detected |
| 03/03 | UGF016 | >10^5^ | *E. coli*, other | 2 | 2 | 16 (I) | R | 31 (S) | S | 26 (S) | S |
| 03/03 | UGF017 | >10^5^ | Gram-positive | 2 | 2 | Not available | Target not detected | Not available | Target not detected | Not available | Target not detected |
| 03/03 | UGF018_1 | No growth | N/A | 2 | 2 | N/A | Target not detected | N/A | Target not detected | N/A | Target not detected |
|  | UGF018_2 |  |  | 2 | 2 |  | Target not detected |  | Target not detected |  | Target not detected |
| 03/03 | UGF019_1 | >10^5^ | *K. pneumoniae* | 2 | 2 | 10 (R) | R | 26 (S) | S | 26 (S) | S |
|  | UGF019_2 |  |  | 2 | 2 |  | R |  | S |  | S |
| 03/03 | UGF020_1 | >10^5^ | *E. coli*, other | 2 | 2 | 0 (R) | Target not detected | 30 (S) | S | 23 (S) | S |
|  | UGF020_2 |  |  | 2 | 2 |  | Target not detected |  | Target not detected |  | S |

**Table S4.** Summary of MCW optimization high microbial load study.

|  | **Number of “Negative” specimens** | **Number of specimens not containing target pathogens** |
| --- | --- | --- |
| Total | 8/36 | 6/36 |
| **Ampicillin** | | |
| Target not detected | | 25/36 |
| Resulting viable specimens | | 11/36 |
| Categorical agreement | | 6/11 = 55% |
| Very major error | | 1/11 = 9% |
| Major error | | 3/11 = 27% |
| Minor error | | 1/11 = 9% |
| **Ciprofloxacin** | | |
| Target not detected | | 22/36 |
| Resulting viable specimens | | 14/36 |
| Categorical agreement | | 14/14 = 100% |
| Very major error | | 0/14 = 0% |
| Major error | | 0/14 = 0% |
| Minor error | | 0/14 = 0% |
| **Sulfamethoxazole-trimethoprim** | | |
| Target not detected | | 23/36 |
| Resulting viable specimens | | 13/36 |
| Categorical agreement | | 13/13 = 100% |
| Very major error | | 0/13 = 0% |
| Major error | | 0/13 = 0% |
| Minor error | | 0/13 |


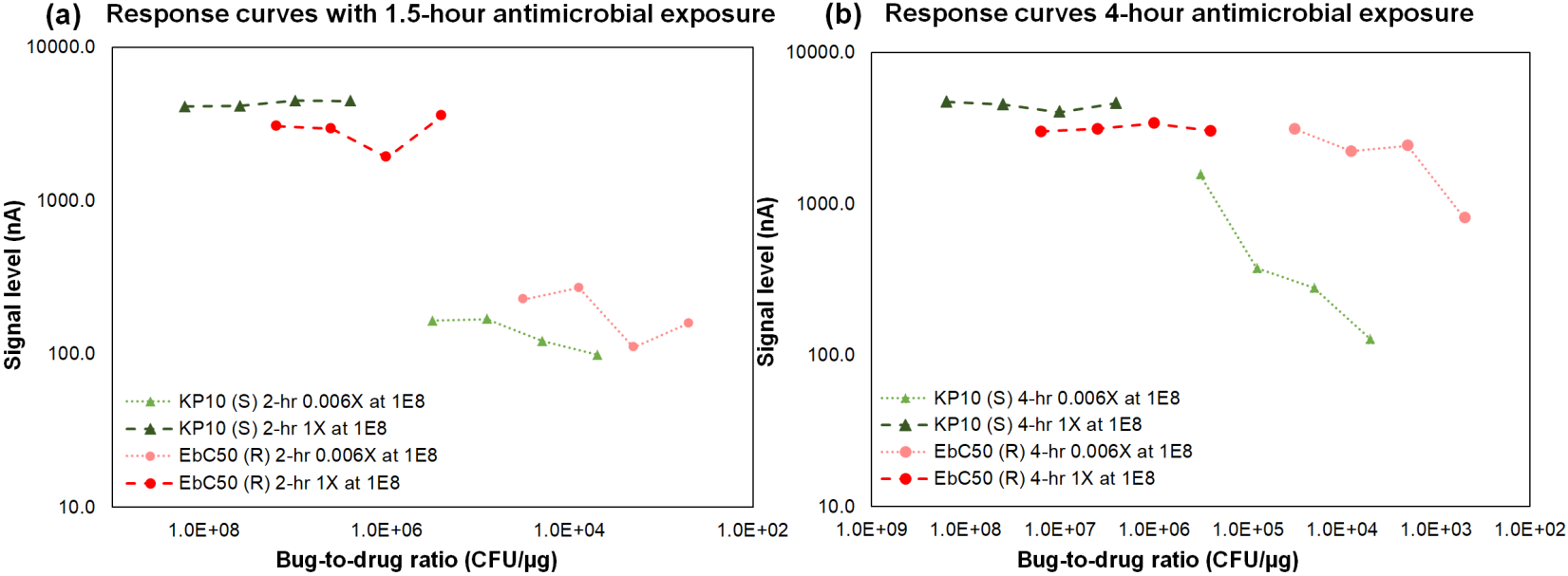


**Figure S5.** Varying antibiotic exposure times to address the limited change in inhibited growth. (a) 10^8^ CFU/mL susceptible and resistant strains tested with 1.5 hours of antibiotic exposure, (b) 10^8^ CFU/mL susceptible and resistant strains tested with 4 hours of antibiotic exposure. Using a 1.5-hour antimicrobial exposure time led to a similar issue in which the susceptible strain was indistinguishable from the resistant strain. The insufficient exposure time resulted in limited growth changes for each strain. Our hypothesis of a longer exposure time leading to more significant changes in growth for the susceptible and resistant strains was confirmed. The 0.06X wells of the susceptible strain demonstrated a decreasing trend, indicative of a susceptible response, and the resistant strain exhibited clear growth across all 0.06X wells, indicating a resistant response to ciprofloxacin. There was no change in signal for the wells containing 1X inoculum due to the use of a high starting inoculum of 10^8^ CFU/mL combined with a 30% enzyme concentration, leading to a capped signal for those wells. With the longer exposure time, the difference between the susceptible and resistant strains was more easily identified.

**Table S5.** Summary of MCW direct-from-specimen AST clinical feasibility study.

| **Ship date** | **MCW specimen** | **Standard of care microbial load (CFU/mL)** | **Standard of care pathogen ID** | **Start volume (mL)** | **Exposure time (hr)** | **AMP disk diffusion** | **AMP direct-from-specimen AST** | **CIP disk diffusion** | **CIP direct-from-specimen AST** | **SXT disk diffusion** | **SXT direct-from-specimen AST** |
| --- | --- | --- | --- | --- | --- | --- | --- | --- | --- | --- | --- |
| 01/07 | GFU011_1 | 2.5×10^4^ | *E. coli*, other | 4 | 3 | N/A | N/A | 33 (S) | Target not detected | N/A | N/A |
|  | GFU011_2 |  |  | 4 | 3 |  |  |  | S |  |  |
| 01/07 | GFU012_1 | 5×10^4^ | *P. mirabilis*, other | 4 | 3 | N/A | N/A | 27 (S) | S | N/A | N/A |
|  | GFU012_2 |  |  | 4 | 3 |  |  |  | S |  |  |
| 01/13 | GFU013_1A | >10^5^ | *K. pneumoniae* | 4 | 3 | N/A | N/A | 29 (S) | S | N/A | N/A |
|  | GFU013_1B |  |  | 2 | 3 |  |  |  | S |  |  |
|  | GFU013_2A |  |  | 4 | 3 |  |  |  | S |  |  |
|  | GFU013_2B |  |  | 2 | 3 |  |  |  | S |  |  |
| 01/13 | GFU014_1A | >10^5^ | *E. coli*, other | 4 | 3 | N/A | N/A | 27 (S) | S | N/A | N/A |
|  | GFU014_1B |  |  | 2 | 3 |  |  |  | S |  |  |
|  | GFU014_2A |  |  | 4 | 3 |  |  |  | S |  |  |
|  | GFU014_2B |  |  | 2 | 3 |  |  |  | Invalid |  |  |
| 01/13 | GFU015_1 | >10^5^ | *E. coli* | 2 | 3 | N/A | N/A | 34 (S) | S | N/A | N/A |
|  | GFU015_2 |  |  | 2 | 3 |  |  |  | S |  |  |
| 01/13 | GFU016_1A | >10^5^ | *E. coli*, other | 4 | 3 | N/A | N/A | 26 (S) | S | N/A | N/A |
|  | GFU016_1B |  |  | 2 | 3 |  |  |  | S |  |  |
|  | GFU016_2A |  |  | 4 | 3 |  |  |  | Invalid |  |  |
| 02/09 | GFU017 | 5×10^4^ | *K. aerogenes*, other | 2 | 3 | N/A | N/A | 27 (S) | S | N/A | N/A |
| 03/30 | UGF021_1 | Not available | Not available | 2 | 2 | 21 (S) | S | 36 (S) | S | 30 (S) | S |
|  | UGF021_2 |  |  | 2 | 2 |  | S |  | S |  | S |
| 03/30 | UGF022_1 | Not available | Not available | 2 | 2 | 20 (S) | Target not detected | 28 (S) | Target not detected | 27 (S) | Target not detected |
|  | UGF022_2 |  |  | 2 | 2 |  | Target not detected |  | Target not detected |  | Target not detected |
| 03/30 | UGF023_1 | Not available | Not available | 2 | 2 | 0 (R) | R | 15 (R) | R | 0/32 (R/S) | Invalid |
|  | UGF023_2 |  |  | 2 | 2 |  | R |  | R |  | Invalid |
| 03/30 | UGF024 | Not available | Not available | 2 | 2 | 18 (S) | S | 30 (S) | S | 26 (S) | S |
| 03/30 | UGF025 | Not available | Not available | 2 | 2 | 20 (S) | S | 33 (S) | S | 23 (S) | S |
| 03/30 | UGF026 | Not available | Not available | 2 | 2 | 18 (S) | S | 34 (S) | S | 27 (S) | S |
| 03/30 | UGF027 | Not available | Not available | 2 | 2 | 30 (S) | Target not detected | 16 (R) | Target not detected | 28 (S) | Target not detected |
| 03/30 | UGF028 | Not available | Not available | 2 | 2 | 18 (S) | S | 35 (S) | S | 26 (S) | S |
| 03/30 | UGF029 | Not available | Not available | 2 | 2 | N/A | Target not detected | N/A | Target not detected | N/A | Target not detected |
| 03/30 | UGF030 | Not available | Not available | 2 | 2 | N/A | Target not detected | N/A | Target not detected | N/A | Target not detected |
| 04/06 | UGF031_1 | Not available | Not available | 2 | 2 | 18 (S) | S | N/A | N/A | 0 (R) | R |
|  | UGF031_2 |  |  | 2 | 2 |  | S |  |  |  | R |
| 04/06 | UGF032_1 | Not available | Not available | 2 | 2 | N/A | Target not detected | N/A | N/A | N/A | Target not detected |
|  | UGF032_2 |  |  | 2 | 2 |  | Target not detected |  |  |  | Target not detected |
| 04/06 | UGF033_1 | Not available | Not available | 2 | 2 | 17 (S) | S | N/A | N/A | 0 (R) | R |
|  | UGF033_2 |  |  | 2 | 2 |  | S |  |  |  | R |
| 04/06 | UGF034_1 | Not available | Not available | 2 | 2 | N/A | Target not detected | N/A | N/A | N/A | Target not detected |
|  | UGF034_2 |  |  | 2 | 2 |  | Target not detected |  |  |  | Target not detected |
| 04/06 | UGF035_1 | Not available | Not available | 2 | 2 | 8 (R) | Target not detected | N/A | N/A | 27 (S) | Target not detected |
|  | UGF035_2 |  |  | 2 | 2 |  | Target not detected |  |  |  | Target not detected |
| 04/06 | UGF036_1 | Not available | Not available | 2 | 2 | N/A | Target not detected | N/A | N/A | N/A | Target not detected |
|  | UGF036_2 |  |  | 2 | 2 |  | Target not detected |  |  |  | Target not detected |
| 04/06 | UGF037_1 | Not available | Not available | 2 | 2 | 25 (S) | R | N/A | N/A | 22 (S) | S |
|  | UGF037_2 |  |  | 2 | 2 |  | R |  |  |  | S |
| 04/06 | UGF038_1 | Not available | Not available | 2 | 2 | 0 (R) | R | N/A | N/A | 23 (S) | S |
|  | UGF038_2 |  |  | 2 | 2 |  | R |  |  |  | S |
| 04/06 | UGF039_1 | Not available | Not available | 2 | 2 | 0 (R) | S | N/A | N/A | 24 (S) | Invalid |
|  | UGF039_2 |  |  | 2 | 2 |  | S |  |  |  | Invalid |
| 04/06 | UGF040_1 | Not available | Not available | 2 | 2 | 21 (S) | Target not detected | N/A | N/A | 25 (S) | Target not detected |
|  | UGF040_2 |  |  | 2 | 2 |  | Target not detected |  |  |  | Target not detected |

**Table S6.** Re-test results of samples called incorrectly during MCW direct-from-specimen AST clinical feasibility study. Samples were re-tested by contriving at the estimated microbial load from the original sample.

| Sample | Organism | Load (CFU/mL) | Starting volume | Exposure time | Specimen dilution | HRP % | Antibiotic | Disk Diffusion (mm) | Direct AST |
| --- | --- | --- | --- | --- | --- | --- | --- | --- | --- |
| GFU014 | *E. coli,* other | 10^6^ | 2 mL | 3 hr | 1X/0.002X | 100 | CIP | 28 (S) | S |
| UGF023 | N/A | 10^8^ | 2 mL | 2 hr | 1X/0.002X | 100 | SXT | 0/30 (R/S) | R/S |
| UGF037 | N/A | 10^8^ | 2 mL | 2 hr | 1X/0.002X | 100 | AMP | 25 (S) | R |
| UGF039 | N/A | 10^5^ | 2 mL | 2 hr | 1X/0.002X | 100 | AMP | 0 (R) | R |

**Table S7.** Susceptibility reporting parameters for AMP results from MCW direct-from-specimen AST clinical feasibility study.

| **Sample** | **Max.drop** | **SW#MaxDrop** | **GC** | **uLoad** | **AccuDrop%** | **Plateau** | **AccuDrop** | **Drop%** | **Determining parameter** |
| --- | --- | --- | --- | --- | --- | --- | --- | --- | --- |
| UGF021_1 | 5644 | 2 | 10000 | 5×10^7^ | 76% | Yes | 7624.7 | 57% | AccuDrop |
| UGF021_2 | 3514 | 2 | 10000 | 5×10^7^ | 55% | No | 5503.2 | 53% | AccuDrop |
| UGF023_1 | 167 | 5 | 10000 | 5×10^7^ | 1% | No | 66.8 | 32% | Drop%+ Accudrop% |
| UGF023_2 | 250 | 5 | 9330.4 | 5×10^7^ | -7% | No | -648.9 | 39% | Drop%+ Accudrop% |
| UGF024_1 | 7552 | 2 | 10000 | 5×10^7^ | 97% | No | 9724.6 | 76% | AccuDrop |
| UGF025_1 | 5987 | 2 | 10000 | 5×10^7^ | 97% | No | 9720.4 | 60% | AccuDrop |
| UGF026_1 | 4883 | 2 | 10000 | 5×10^7^ | 90% | Yes | 8963.8 | 49% | AccuDrop |
| UGF028_1 | 3459 | 2 | 10000 | 5×10^7^ | 19% | No | 1915.1 | 35% | Drop%+ Accudrop% |
| UGF031_1 | 2224 | 1 | 2299.2 | 5×10^5^ | 79% | Yes | 1816.6 | 97% | Drop%+ Accudrop% |
| UGF031_2 | 2348 | 1 | 3907.9 | 5×10^5^ | 76% | Yes | 2953.5 | 60% | AccuDrop |
| UGF033_1 | 2655 | 3 | 10000 | 5×10^7^ | 77% | No | 7705.2 | 27% | AccuDrop |
| UGF033_2 | 2899 | 3 | 10000 | 5×10^7^ | 80% | No | 8018.7 | 29% | AccuDrop |
| UGF037_1 | 1642 | 1 | 10000 | 5×10^7^ | 4.93% | No | 492.9 | 16% | Drop%+ Accudrop% |
| UGF037_2 | 3049 | 2 | 10000 | 5×10^7^ | 4.97% | No | 497.4 | 35% | Drop%+ Accudrop% |
| UGF038_1 | 431 | 6 | 10000 | 5×10^7^ | 3% | Yes | 275.6 | 31% | Accudrop% |
| UGF038_2 | 1499 | 2 | 9502.7 | 5×10^7^ | -3% | No | -246.0 | 15% | Drop%+ Accudrop% |
| UGF039_1 | 324 | 1 | 751.9 | 5×10^4^ | 51% | No | 383.9 | 43% | Drop%+ Accudrop% |
| UGF039_2 | 208 | 1 | 935.5 | 5×10^4^ | 41% | No | 382.1 | 22% | Drop%+ Accudrop% |
| UGF037_retest | 2133 | 2 | 10000 | 5×10^7^ | 2% | Yes | 239.9 | 24% | Accudrop% |
| UGF039_retest | 31 | 1 | 190.3 | 5×10^4^ | 21% | No | 40.1 | 16% | Drop%+ Accudrop% |

**Table S8.** Susceptibility reporting parameters for CIP results from MCW direct-from-specimen AST clinical feasibility study.

| **Sample** | **Max.drop** | **SW#MaxDrop** | **GC** | **uLoad** | **AccuDrop%** | **Plateau** | **AccuDrop** | **Drop%** | **Determining parameter** |
| --- | --- | --- | --- | --- | --- | --- | --- | --- | --- |
| UGF021_1 | 1656 | 5 | 10000 | 1.6×10^9^ | 20% | No | 1982 | 81% | Drop%+ Accudrop% |
| UGF021_2 | 4844 | 2 | 10000 | 1.6×10^9^ | 75% | Yes | 7505 | 48% | Accudrop |
| UGF023_1 | 120 | 6 | 10000 | 1.6×10^9^ | 3% | No | 280 | 30% | Accudrop% |
| UGF023_2 | 412 | 6 | 10000 | 1.6×10^9^ | 8.4% | Yes | 837 | 39.9% | Accudrop% |
| UGF024_1 | 1501 | 3 | 10000 | 1.6×10^9^ | 20% | No | 2019 | 15% | Drop%+ Accudrop% |
| UGF025_1 | 7534 | 1 | 10000 | 1.6×10^9^ | 90% | No | 8989 | 75% | Drop%+ Accudrop% |
| UGF026_1 | 542 | 5 | 10000 | 1.6×10^9^ | 6% | No | 615 | 82% | Accudrop |
| UGF028_1 | 737 | 5 | 10000 | 1.6×10^9^ | 8% | Yes | 801 | 87% | Accudrop% |
| GFU011_2 | 92 | 1 | 122 | 1.6×10^6^ | 82% | Yes | 101 | 75% | Drop%+ Accudrop% |
| GFU012_1 | 1138 | 1 | 1230 | 1.6×10^6^ | 94% | Yes | 1157 | 93% | Drop%+ Accudrop% |
| GFU012_2 | 353 | 1 | 522 | 1.6×10^6^ | 94% | No | 491 | 68% | Drop%+ Accudrop% |
| GFU013_1A | 469 | 5 | 10000 | 1.6×10^9^ | 11% | No | 1114 | 32% | Drop%+ Accudrop% |
| GFU013_1B | 359 | 5 | 10000 | 1.6×10^9^ | 8.2% | No | 821 | 36% | Drop%+ Accudrop% |
| GFU013_2A | 3325 | 3 | 10000 | 1.6×10^9^ | 65% | No | 6450 | 33% | Accudrop |
| GFU013_2B | 3753 | 2 | 10000 | 1.6×10^9^ | 45% | No | 4495 | 38% | Accudrop |
| GFU014_1A | 2166 | 5 | 2645 | 1.6×10^7^ | 148% | No | 3921 | 98% | Accudrop |
| GFU014_1B | 469 | 1 | 1666 | 1.6×10^7^ | 39% | No | 643 | 28% | Drop%+ Accudrop% |
| GFU014_2A | 156 | 1 | 1666 | 1.6×10^7^ | 46% | No | 142 | 50% | Drop%+ Accudrop% |
| GFU015_1 | 715 | 1 | 1043 | 1.6×10^6^ | 76% | Yes | 797 | 69% | Drop%+ Accudrop% |
| GFU015_2 | 634 | 1 | 876 | 1.6×10^6^ | 78% | Yes | 683 | 72% | Drop%+ Accudrop% |
| GFU016_1A | 1072 | 1 | 2495 | 1.6×10^7^ | 89% | Yes | 2231 | 43% | Accudrop |
| GFU016_1B | 639 | 1 | 1554 | 1.6×10^7^ | 103% | Yes | 1599 | 41% | Drop%+ Accudrop% |
| GFU017 | 3245 | 5 | 10000 | 1.6×10^9^ | 34% | Yes | 3418 | 32% | Accudrop |
| GFU014_retest | 4242 | 1 | 4512 | 1.6×10^8^ | 99% | No | 4469 | 94% | Accudrop |

**Table S9.** Susceptibility reporting parameters for SMZ-TMP results from MCW direct-from-specimen AST clinical feasibility study.

| **Sample** | **Max.drop** | **SW#MaxDrop** | **GC** | **uLoad** | **AccuDrop%** | **Plateau** | **AccuDrop** | **Drop%** | **Determining parameter** |
| --- | --- | --- | --- | --- | --- | --- | --- | --- | --- |
| UGF021_1 | 1672 | 5 | 10000 | 4×10^8^ | 17% | No | 1686 | 91% | Drop%+ Accudrop% |
| UGF021_2 | 2691 | 2 | 10000 | 4×10^8^ | 51% | No | 5080 | 27% | AccuDrop% |
| UGF023_1 | 62 | 5 | 9868 | 4×10^8^ | 1% | No | 102 | 13% | AccuDrop |
| UGF023_2 | 2943 | 3 | 10000 | 4×10^8^ | 34% | No | 3360 | 26% | AccuDrop |
| UGF024_1 | 98 | 5 | 10000 | 4×10^8^ | 1% | No | 105 | 56% | Drop%+ Accudrop% |
| UGF025_1 | 7059 | 1 | 10000 | 4×10^8^ | 73% | No | 7309 | 71% | AccuDrop |
| UGF026_1 | 388 | 5 | 10000 | 4×10^8^ | 4% | No | 418 | 77% | Drop%+ Accudrop% |
| UGF028_1 | 662 | 5 | 10000 | 4×10^8^ | 7% | No | 741 | 75% | Drop%+ Accudrop% |
| UGF031_1 | 1251 | 1 | 4104 | 4×10^7^ | 41% | Yes | 1668 | 30% | Drop%+ Accudrop% |
| UGF031_2 | 624 | 1 | 3094 | 4×10^6^ | 30% | No | 918 | 20% | Drop%+ Accudrop% |
| UGF033_1 | 906 | 5 | 10000 | 4×10^8^ | 15% | No | 1524 | 17% | Drop%+ Accudrop% |
| UGF033_2 | 707 | 5 | 10000 | 4×10^8^ | 4.5% | No | 455 | 15% | AccuDrop% |
| UGF037_1 | 736 | 5 | 10000 | 4×10^8^ | 8% | No | 812 | 74% | Drop%+ Accudrop% |
| UGF037_2 | 1714 | 5 | 10000 | 4×10^8^ | 19% | No | 1872 | 85% | Drop%+ Accudrop% |
| UGF038_1 | 1123 | 5 | 10000 | 4×10^8^ | 15% | No | 1458 | 66% | Drop%+ Accudrop% |
| UGF038_2 | 501 | 5 | 10000 | 4×10^8^ | -4% | No | -434 | 59% | Drop%+ Accudrop% |


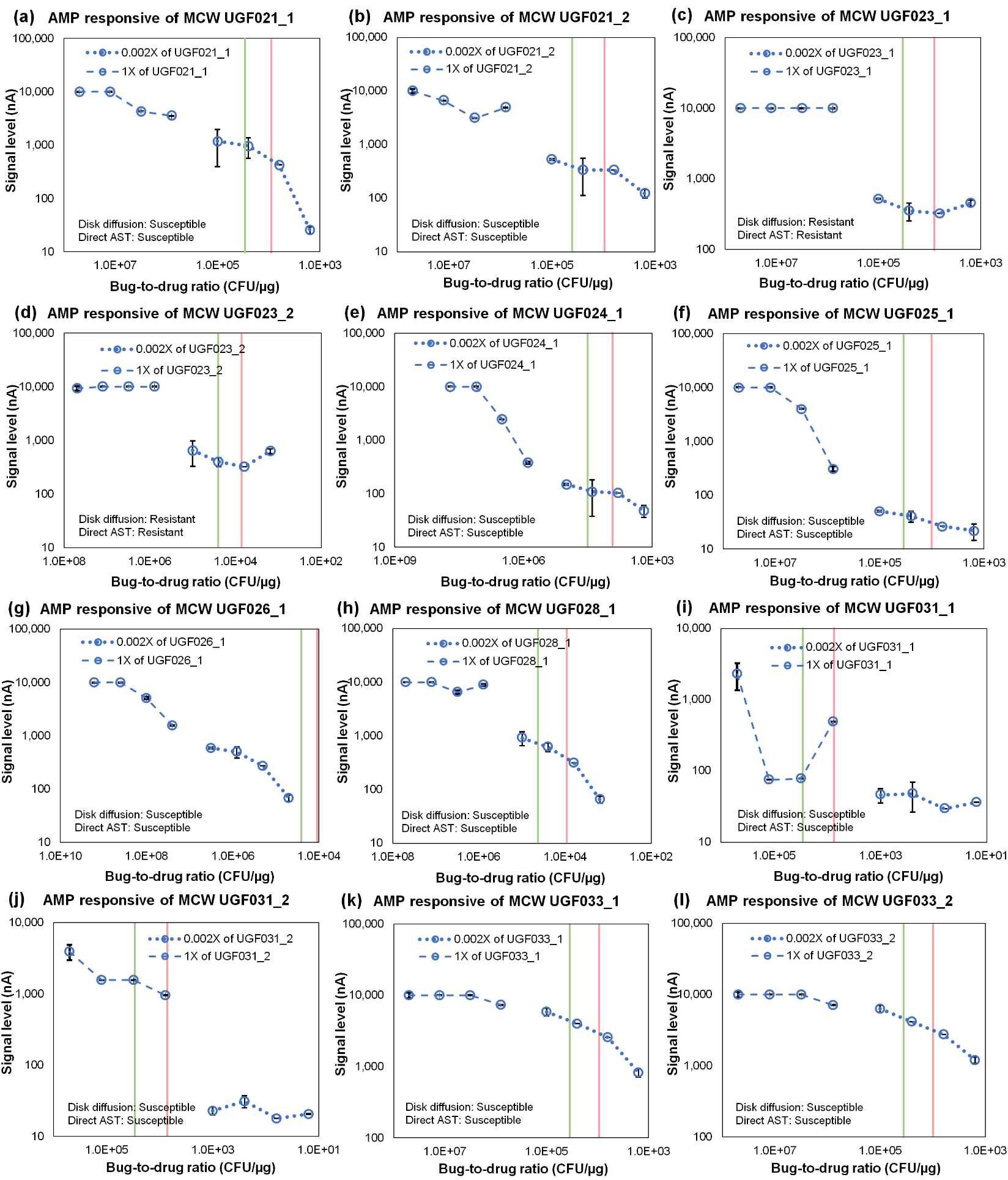


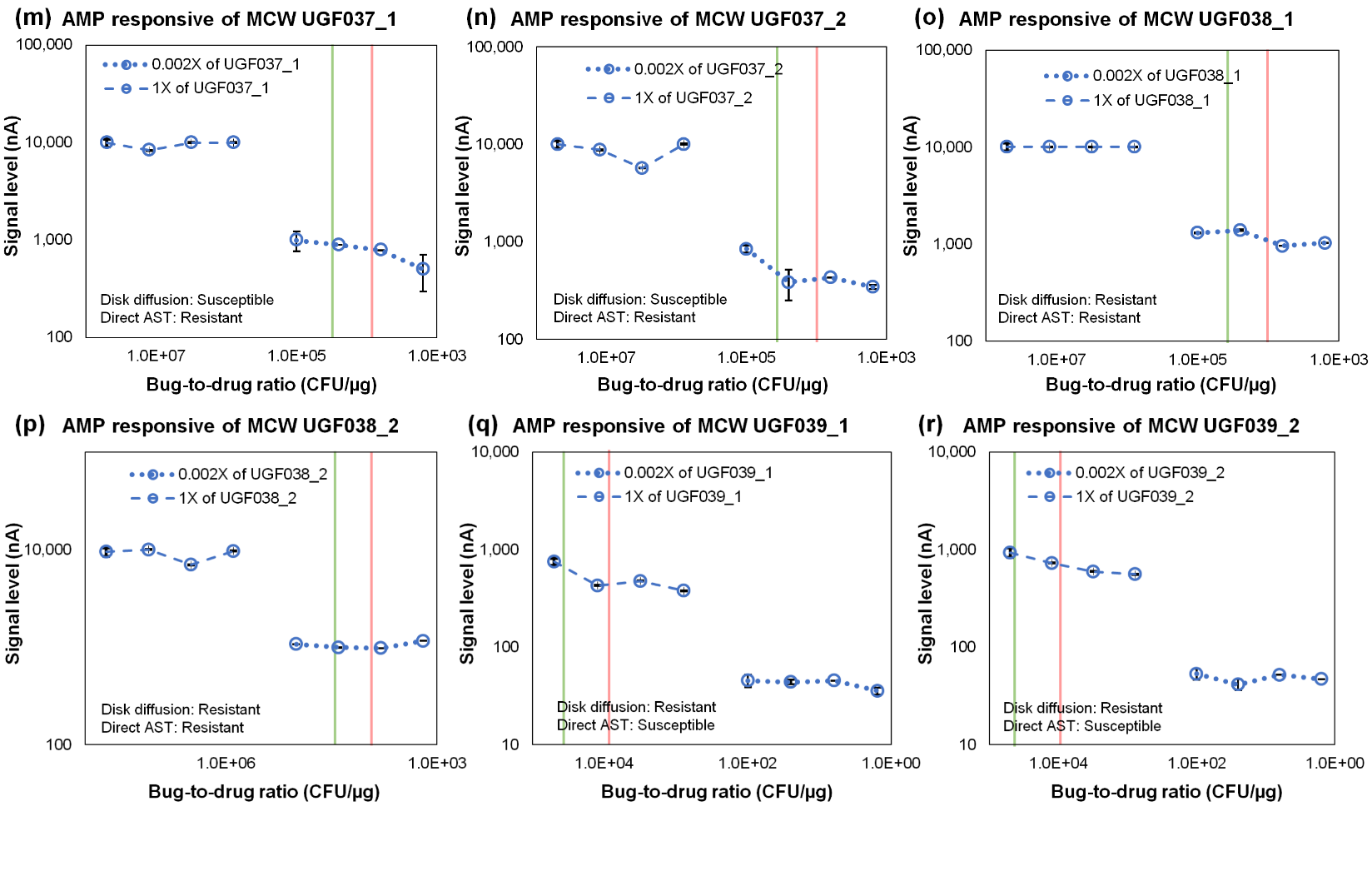


**Figure S6.** Ampicillin response curves for MCW shipping specimens reported positive. Susceptible and resistant breakpoint bug-to-drug ratios indicated by green and red vertical lines, respectively.


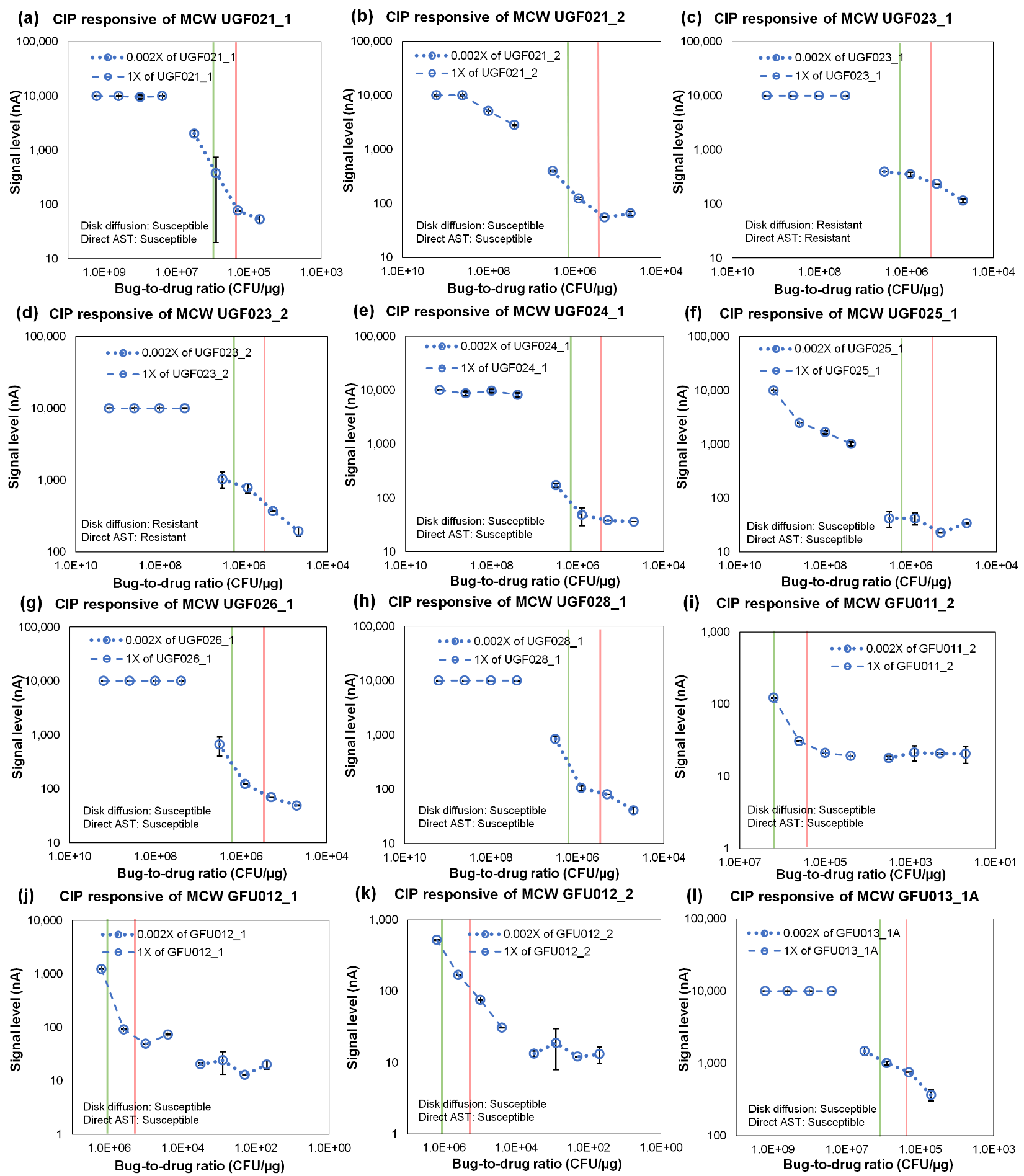


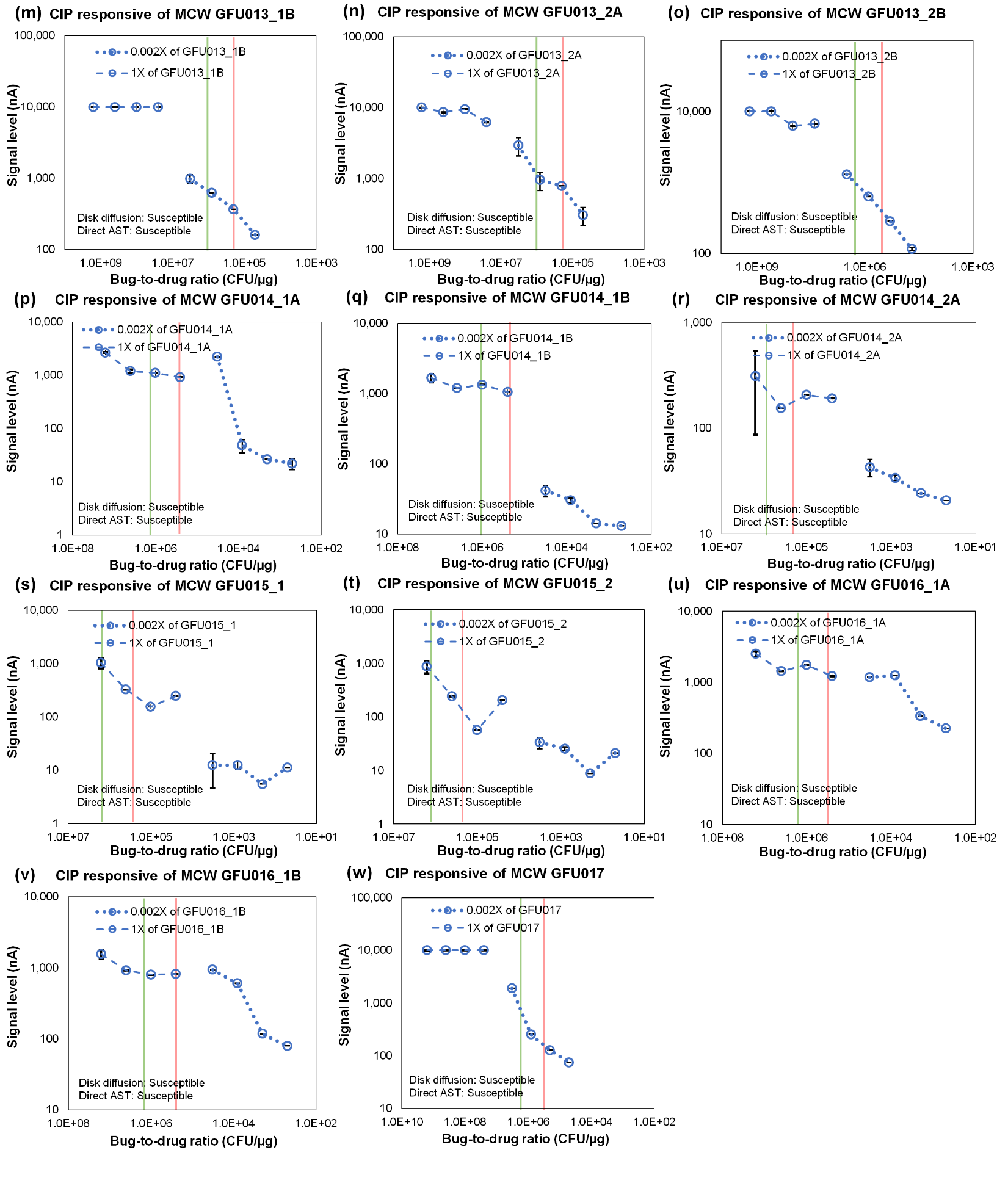


**Figure S7.** Ciprofloxacin response curves of MCW shipping specimens reported positive. Susceptible and resistant breakpoint bug-to-drug ratios indicated by green and red vertical lines, respectively.


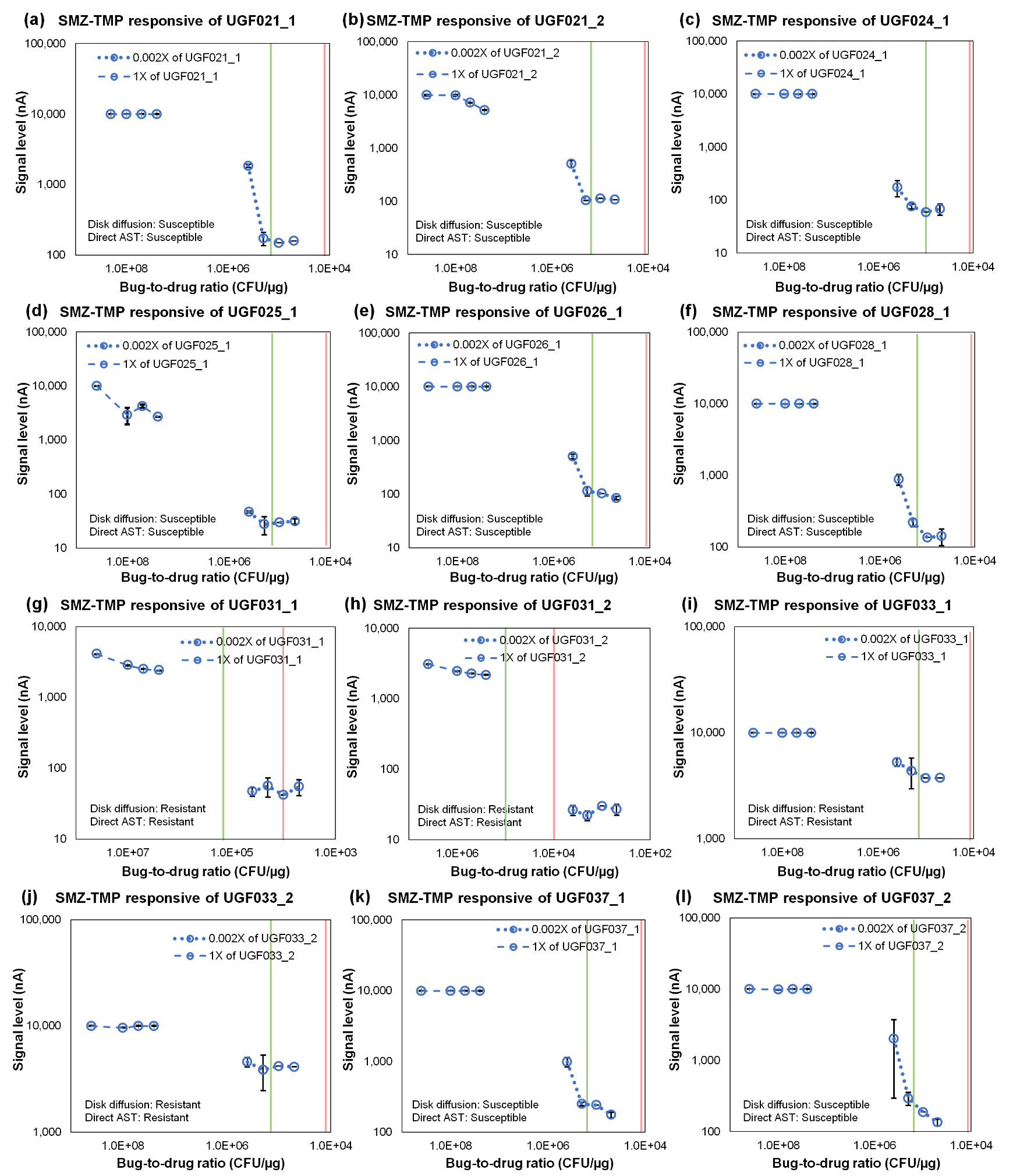


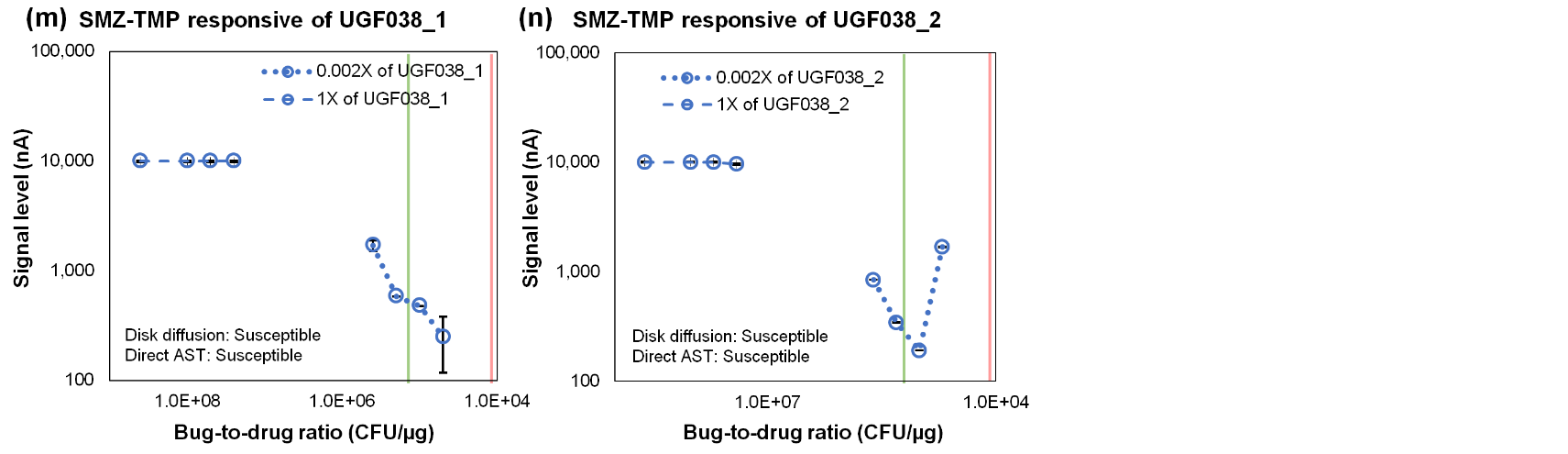


**Figure S8.** Sulfamethoxazole-trimethoprim response curves of MCW shipping specimens reported positive. Susceptible and resistant breakpoint bug-to-drug ratios indicated by green and red vertical lines, respectively.
